# Evaluation of transgenic chickpea harboring codon-modified *Vip3Aa* against gram pod borer (*Helicoverpa armigera* H.)

**DOI:** 10.1371/journal.pone.0270011

**Published:** 2022-06-24

**Authors:** Prateek Singh, Sujayanand G. K., Shallu Thakur, Meenal Rathore, Om Prakash Verma, Narendra Pratap Singh, Alok Das

**Affiliations:** 1 Division of Plant Biotechnology, ICAR-Indian Institute of Pulses Research, Kanpur, Uttar Pradesh, India; 2 Department of Molecular and Cellular Engineering, Jacob Institute of Biotechnology and Bioengineering, SHUATS, Prayagraj, Uttar Pradesh, India; 3 Division of Crop Protection, ICAR-Indian Institute of Pulses Research, Kanpur, Uttar Pradesh, India; University of Texas at Dallas, UNITED STATES

## Abstract

The gram pod borer is a major pest of chickpea, accounting for average annual yield losses to the tune of 40–50%. VIP3Aa, a class of insecticidal protein with different receptor binding site in the insect’s midgut compared to *Bt*-crystal protein, offers an alternative protection strategy against Lepidopteran insects. Here, we report evaluation of genetically engineered chickpea lines harboring codon modified *Vip3Aa* (cm*Vip3Aa*) against the Lepidopteran insect pest, gram pod borer. The synthetic codon modified, cm*Vip3Aa* gene of 2,370 bp was sub-cloned in modified plant expression vector and used for direct transformation of embryonic axis explants of chickpea (*cv*. DCP 92–3), with transformation efficiency of 4.30%. Presence and transmission of transgene across two generations were confirmed by PCR and Southern blot analyses in the five selected transgenic chickpea lines. Real Time PCR analyses indicated variable levels of cm*Vip3Aa* expression in the transgenic chickpea lines (average Cq values 15.01±0.86 to 19.32±0.10), which were absent in the non-transgenic counterpart. Detached leaf insect bioassay indicate larval mortality (up to 39.75%), reduced larval feeding (up to 82.91%) and reduced larval weight gain (up to 68.23%), compared to control lines. Evaluation of gene offers a platform to identify efficacious insecticidal gene that can be used for insect resistance management in chickpea.

## Introduction

Chickpea (*Cicer arietinum* L.) is a major class of grain legume, cultivated on 13.71 mha land in more than 55 countries of the world with an annual production of 14.25 mt [1]. India is the largest producer of chickpea with 11.9 mt accounting for more than two-third of world’s production [2]. Chickpea grain and other value-added products are cheap source of dietary protein, minerals and are considered as poor man’s meat among the vegetarian population of the world. Despite its nutritional importance and soaring global demand, its productivity has stagnated since the last decade with average annual production <1.0 t in India. Global chickpea production is hampered due to various biotic and abiotic stresses encountered during the crop cycle and post-harvest loss during storage. Amongst the various biotic stresses, gram pod borer (*Helicoverpa armigera* Hubner) is the most devastating insect pest in the field accounting for yield losses on all major ecologies of the world. There were three major pod borer outbreaks during last decade, resulting in 10–80% yield losses due to pod damage [3]. Worldwide, economic loss to the tune of US $ 2.0 billion annually has been projected, despite using insecticides costing over US $ 1.0 billion [4].

The early instar stage larvae of the insect preferentially devour on chickpea leaves and tender twigs, while the adult larvae feed on flowers and pods resulting in the yield losses ranging from 40–50% [5]. Conventional pest management techniques against this pest have fallen short due to its polyphagous feeding habit, high fecundity, and ability to evolve rapidly against insecticides [6,7]. Limited availability of insect resistant traits among the wild genotypes of chickpea and sexual incompatibility are the major constraints in the development of insect resistant varieties through conventional breeding. Recently, QTLs explaining phenotypic variance for pod borer resistance component traits were reported in chickpea [8]. The insecticidal *Bacillus thuringiensis* (*Bt*) gene has been reported to be completely effective against this pest and transgenic chickpea harboring class I crystal (*Cry*) gene and various modifications have been reported [3,9–16]. However, single gene based resistance may not sustain in a long run, particularly in Indian context, where first generation of Bt-cotton (BOLGARD I) are being grown extensively. Hence, the current study was taken up to evaluate the efficacy of Vip3Aa against gram pod borer, besides Cry1Ac (Bt gene). Combination of Cry1 and effective Vip gene should confer broader control against gram pod borer.

The Vegetative Insecticidal Protein (VIP) is a class of insecticidal *Bt*-toxins that are secreted naturally by various entomopathogenic bacteria during the vegetative growth phase. These proteins are classified into four families–VIP1, VIP2, VIP3 and VIP4 [17]; based on their amino acid groups present. While VIP1 (100kDa) and VIP2 (52kDa) are binary toxins and reported to be effective against some members of Coleoptera and Hemiptera [18], the third member of the class, VIP3 (88.5 kDa) are single chain insecticidal proteins that are unique (no sequence similarity with VIP1 and VIP2) and are found effective against Lepidopteran insects [19–21]. Current understanding suggests that VIP3A toxin confers resistance against lepidopteran insects by specifically binding to their brush border membrane vesicles (BBMV) leading to formation of ion channel that selectively damages their midgut lining [22,23]. However, further research is needed to elucidate its mode of action.

VIP3 in its native state is produced as an inactive protoxin (88.5 kDa) that is activated by the protease activity of digestive enzymes present in the insect midgut leading to cell death. Activated VIP3Aa toxin binds to Sf-SR-C (scavenger receptor) and Fgfr (fibroblast growth receptor) receptors found in the insect’s midgut [24,25]. The protoxin in the native state consist of five domains, starting from N-terminal to the primary protease cleavage site (K198) [26,27]. The second domain is present in the region between 200–325 amino acid residues immediately after the cleavage site (exposed loop connecting α4 and α5 helices) of protoxin and contains five α helices (α1-α5) and a tetrameric core. Third domain is present in the region between 328–532 amino acid residues and contains three antiparallel β-sheet forming β-prism similar to the one present in CRY δ-endotoxin. It is reported to be involved in the cell binding of VIP3A toxin. Domain fourth is the region between 537–667 amino acid residues and the fifth domain is in the region between 679–789 amino acid residues. Both domain 4 and domain 5 are glycan binding motifs entirely consisting of β-sheet fold forming “jelly-roll” topology [28,29]. Currently, there are no reports of expression of *Vip3Aa* gene in chickpea and their efficacy studies in controlling the insect pest, gram pod borer.

In the present investigation, synthetic codon modified cm*Vip3Aa* gene cloned in a plant expression vector was utilized for direct genetic transformation (particle gun bombardment) in a well-adapted *desi* chickpea cultivar, DCP 92–3. Five cm*Vip3Aa* expressing independent chickpea lines were characterized based on presence and expression across two generations followed by insect bioassay of the developed transgenic chickpea lines. This is the maiden report of development of transgenic chickpea harboring cm*Vip3Aa* and their efficacy testing against target insect pest, gram pod borer.

## Materials and methods

### Codon modification and *in silico* characterization of *Vip3Aa*

Native gene sequence of *Vip3Aa* was retrieved from NCBI database *(*Accession Number L48811) and codon modified according to the Kazusa database (https://www.kazusa.or.jp/codon/), in addition to removal of RNA destabilization elements for expression in chickpea. The codon modified gene sequence (cm*Vip3Aa*) and the translated protein sequence (cmVIP3Aa) were aligned to identify changes in the modified sequence (https://www.ebi.ac.uk/Tools/msa/CLUSTALO/) [30]. Protein-protein interactions were analyzed for the cmVIP3Aa and native chickpea proteins using STRING 11.0 (https://www.expasy.org/resources/string). The allergenicity potential of the VIP3Aa protein was determined using AllergenOnline version 21.0 (http://www.allergenonline.org/) [31,32]. Post structural, functional and stability analysis, the modified sequence was submitted to the NCBI database (https://www.ncbi.nlm.nih.gov/).

### Sub-cloning of *cmVip3Aa* and genetic transformation

The codon modified cm*Vip3Aa* gene was synthesized using GeneMaker^®^ Multi-Technology platform (Eurofins Genomics India Pvt. Ltd, India) and sub-cloned in modified expression vector, pRI201-AN (Takara Bio Inc., Japan) using *Nde*I and *Sal*I (NEB Inc. UK) restriction sites). The native plant expression vector pRI201-AN, harbor an alcohol dehydrogenase (ADH) gene-derived 5′untranslated region (5′-UTR) (translational enhancer region) downstream of the 35S promoter for driving cm*Vip3Aa* expression in plants and an HSP fused termination sequence for transcription termination. The plant antibiotic selection marker, neomycin phosphotransferase II (*npt*II) was eliminated using *Kpn*I and *MauB*I (NEB Inc. UK). The modified vector was confirmed by restriction digestions and Sanger sequencing [33].

Breeders’ seed of *desi* chickpea cultivar, DCP 92–3 was used for genetic transformation experiments. Intact embryonic axis was used as explants for direct genetic transformation using Biolistic Particle Delivery System-1000/He as described earlier [34]. Embryonic axis derived plantlets exhibiting developed roots were transferred on soil matrix and established to maturity in the Transgenic Containment Facility (PBSL1). Selfed seeds of primary transformants were harvested from mature fertile plants and were employed for generation advancement.

### PCR screening and Southern blot analyses

The presence and transmission of the transgene in the established transgenic chickpea lines and selfed progenies were confirmed as described earlier [35]. The presence of cm*Vip3Aa* gene in transgenic chickpea plants were confirmed by PCR using the gene specific primers [VN4F: 5′ATCAGCAAGACCAAGAAGCTTTCTAC3′ (forward) and VN4R:5′ACTGATACTGGTGGTGATCTTACTC3′ (reverse)] using the following thermal profile: initial denaturation at 94°C for 2 min, followed by 35 cycles of denaturation at 94°C for 30 s, annealing at 60°C for 30 s and extension at 72°C for 60 s, with a final extension at 72°C for 10 min. PCR products were resolved in 1.0% agarose gel stained with Ethidium Bromide (EtBr) in 1X TAE buffer.

Transformation efficiency was estimated based on presence and transmission of transgene to progenies (T_1_), using the formula:

$$Transformation\;efficiency\;(\%)=\frac{No.of\;PCR\;positive\;lines}{Total\;No.of\;explants\;bombarded}\times 100$$

where, No. of PCR positive lines indicate number of primary transformants (T_0_) from which PCR positive (T_1_) progenies could be identified. Segregation ratio was calculated based on PCR screening of the progenies (T_1_) derived from individual T_0_. Chi-square (χ^2^) test was conducted for understanding segregation pattern in five selected lines.

**Fig 1:**
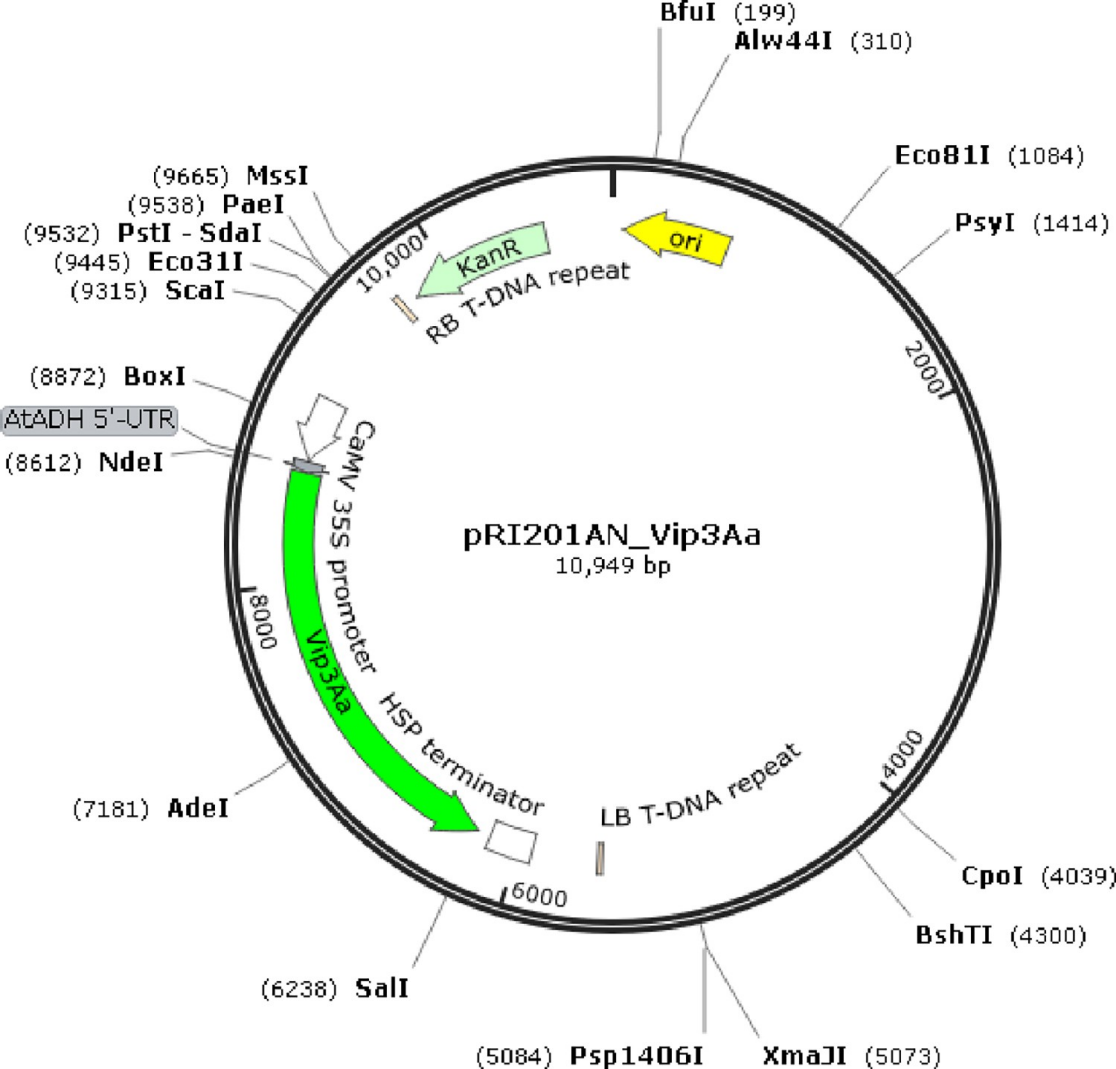
Modified plant expression vector pRI 201-AN harboring cm*Vip3Aa*.

For genomic Southern hybridization, DNA was isolated from leaves, pooled from PCR positive T_2_ progenies of six transgenic lines (VPS13, VPS14, VPS47, VPS57, VPS66 and VPS77) and control (DCP 92–3). Isolated DNA was digested with restriction endonuclease, *Sal*I (restricting at cm*Vip3Aa* terminal) and double-digested with *Nde*I and *Sal*I (releasing cm*Vip3Aa*) separately (Refer Fig 1). The digested products were blotted onto positively charged Nylon Membranes and hybridized with DIG labeled probe (783 bp).The hybridization signal was detected using colorimetric substrate NBT/BCIP tablets (Roche Diagnostics GmbH, Germany/ Sigma-Aldrich, USA). Further, T_2_ progenies derived from PCR positive T_1_ plants (from five tested lines) were screened for understanding the organization of the transgene (zygosity) based on PCR analysis.

### Reverse Transcriptase-PCR, quantitative Real Time-PCR and ELISA

Total RNA was extracted from leaves (~100 mg) of T_2_ progenies of five transgenic lines (VPS14, VPS47, VPS57, VPS66 and VPS77) and control plants (DCP 92–3), using Spectrum^TM^ Plant total RNA Kit (Sigma, USA). The cDNA first-strand was synthesized using RevertAid First Strand cDNA Synthesis Kit (Thermo Scientific, USA). PCR amplification of cDNA was performed using specific primers of a cmVip3Aa [RTVF: 5′CTGGTGGTGATCTTACTC3′ and RTVR: 5′CACCATCAAGCTTACCAG3′] and internal control eukaryotic initiation factor 4α (IF4α) genes [IF4aF: 5′TGGACCAGAACAC TAGGGACATT3′ (forward); IF4aR:5′AAACACGGGAAGACCCAGAA3′ (reverse)] and amplification documented.

The quantitative Real-Time PCR (qRT-PCR) was performed using PowerUp^TM^ SYBR^TM^ green master mix (ThermoFisher Scientific, USA) in ArialMX real-time (qPCR) instrument (Agilent Technologies, USA). The specificity of the PCR amplification was checked with a heat dissociation curve (60–95°C) following the final cycle of the PCR. Quantitative variation among different samples was determined using the ΔΔ*C*q method and all the data were analyzed using in-built Agilent AriaMX software (Agilent Technologies, USA). The mean value for the expression level of the gene was calculated from three independent experiments, for all the five transgenic chickpea lines.

Expression of cmVIP3Aa protein in leaves and pod wall of the transgenic chickpea T_2_ progenies were detected post flowering (106 Days After Sowing) stages for tissue specific expression studies using qualitative Vip3Aa ELISA kit (Agdia Inc., USA). Total soluble protein (TSP) was isolated from leaves and pod walls of all the five transgenic progenies and control, and quantified using Bradford assay [36]. The absorbance was measured at 450 nm wavelength in ELISA reader (Multiskan EX–Thermo Fisher Scientific, USA), and a comparative histogram was plotted. Regression analysis was conducted to understand the effect of leaf cmVIP3Aa protein absorbance (450 nm) and larval mortality, using MS-Excel software (Microsoft Corporation, USA).

### Insect bioassay

Insect bioassays were performed in 3–5 selected transgenic chickpea plants from all five lines as described earlier [35]. Ten chickpea leaflets were clipped from each plant for the five transgenic lines {VPS14 (5 plants), VPS47 (3 plants), VPS57 (3 plants), VPS66 (3 plants) and VPS77 (4 plants) (variation among the number of chickpea plants screened is based on the availability of healthy twigs for detached leaf insect bioassay)} at T_2_ stages and similarly in case of non-transgenic control (DCP 92–3), 4 chickpea plants were subjected for screening. The age of plants at the time of screening was ~ 90 days after sowing; the leaf twigs were surface sterilized with 0.01% NaOCl to remove any inert material/dust adhering to it. Initial weights of leaflets were recorded and placed in a 50 ml container having 2% water agar supplemented with 0.1% (w/v) sorbic acid. The freshly hatched cohort of 3^rd^ instar stage larvae of gram pod borer, *H*. *armigera* reared on artificial diet were used for insect bioassay. To each container, single larvae was released carefully on the chickpea leaflet after recording the initial weight of larva, and the containers were placed in BOD incubator at 25±5°C and 70±5% relative humidity. A total of 10 larvae (1 larvae/leaflet) were used for assessing the insect mortality potential of each plant. The entire experiment was conducted in completely randomized design (CRD) and experiments were repeated thrice. Percent mortality was calculated from the detached leaf insect bioassay data recorded for 24 to 168 h after larva release [37]. After 7 days, the remaining leaflet weight were recorded for calculating the amount of leaf consumed by individual larva and the average leaf weight consumed per plant were calculated. Similarly, the final larval weights were recorded for the live larva for each plant on the 7^th^ day to calculate the larval weight gain. The percentage reduction in leaf weight consumed over control and percentage decrease in larval weight gain over control was calculated by using the formula given below:

$$Percentage\;reduction\;in\;leaf\;weight\;consumed\;over\;control=\{\frac{Leaf\;weight\;consumed\;per\;larva\;per\;plant\;in\;control-Leaf\;weight\;consumed\;per\;larva\;per\;plant\;in\;treatment}{Leaf\;weight\;consumed\;in\;control\;per\;larva\;per\;plant}\}$$

$$Percentage\;decrease\;in\;larva\;weight\;gain\;over\;control=\left\{\frac{Larva\;weight\;gain\;per\;larva\;per\;plant\;in\;control-Larva\;weight\;gain\;per\;larva\;per\;plant\;in\;treatment}{Larva\;weight\;gain\;in\;control\;per\;larva\;per\;plant}\right\}x\;100$$

The percent mortality data, percentage reduction in leaf weight consumed over control and percentage decrease in larval weight gain over control were subjected to statistical analysis in SAS 6.2 (SAS Institute, Inc., Cary, NC, USA) by using ANOVA procedure with *post hoc* test Duncan’s multiple range test (DMRT). P < 0.01 was considered to be statistically significant.

## Results

### Bioinformatics analysis of *cmVip3Aa* gene

Codon modification of *Vip3Aa* as per Kazusa database resulted in decrease in total purine content and increase in pyrimidines content. Total purine: adenine (A) and guanine (G) content decreased from 38% to 30% and from 18% to 16% respectively, while the pyrimidines: thymine (T) and cytosine (C) content increased from 32% to 40% and from 12% to 14% respectively compared to the original reported sequence. Potential eukaryotic transcription termination signals were replaced for expression in plants. The cm*Vip3Aa* sequence of 2,370 bp encodes 789 amino acids indicated 79.49% sequence identity between the native *Vip3Aa* and the modified cm*Vip3Aa* gene, without any change in the amino acid sequences (S1 and S2 Info). No protein-protein interaction could be retrieved between the cm*Vip3Aa* and chickpea proteins, documented at STRING dataset. The results of allergen search of Vip3Aa protein using AllergenOnline database did not identify significant alignment with any of the known allergens. The maximum percentage identity found was less than 50% (22.8% identity with tropomyosin in 127 amino acid overlap) and high statistical expectation score (E score) of 0.17 for Full FASTA36 bases analysis. The statistical-fit histogram showed only minor deviation from the expected distribution of alignments. No match was detected using Sliding 80mer Window 36 and 8mer Exact Match. Modified cm*Vip3Aa* sequence was submitted to GenBank, NCBI (Accession No. MZ130099).

### Development of transgenic chickpea with *cmVip3Aa*

Synthesized cm*Vip3Aa* was sub-cloned in the modified pRI201-AN (8.6 kb) vector, by directional approach between the *Nde*I and *Sal*I restriction sites (Fig 1). The recombinant vector harboring cm*Vip3Aa* was confirmed by restriction digestion (S1 Fig) and Sanger sequencing.

Embryonic axis explants were bombarded using modified plant expression vector harboring insecticidal cm*Vip3Aa*. A total of 4,187 chickpea embryonic axis explants were bombarded and 2560 *in-vitro* developed putative transformed chickpea plantlets (T_0_) could be successfully established to mature fertile plants in the Transgenic Containment Facility (PBSL-1). The seeds collected from the T_0_ progenies were screened in the subsequent generations (T_1_ and T_2_). A total 180 (T_0_) transgenic chickpea lines were identified to harbor and transmit the cm*Vip3Aa* gene in their genome based on PCR screening at T_1_ stage. The entire direct transformation system adopted in the study is depicted (S2 Fig). Selfed seeds were harvested and employed for generation advancement (T_1_ and T_2_) and molecular analyses of the progenies derived thereof, subsequently. Here, we report generation advancement and segregation analysis of five transgenic chickpea lines *viz*. VPS14, VPS47, VPS57, VPS66 and VPS77.

### Molecular characterization of transgenic chickpea

**Fig 2:**
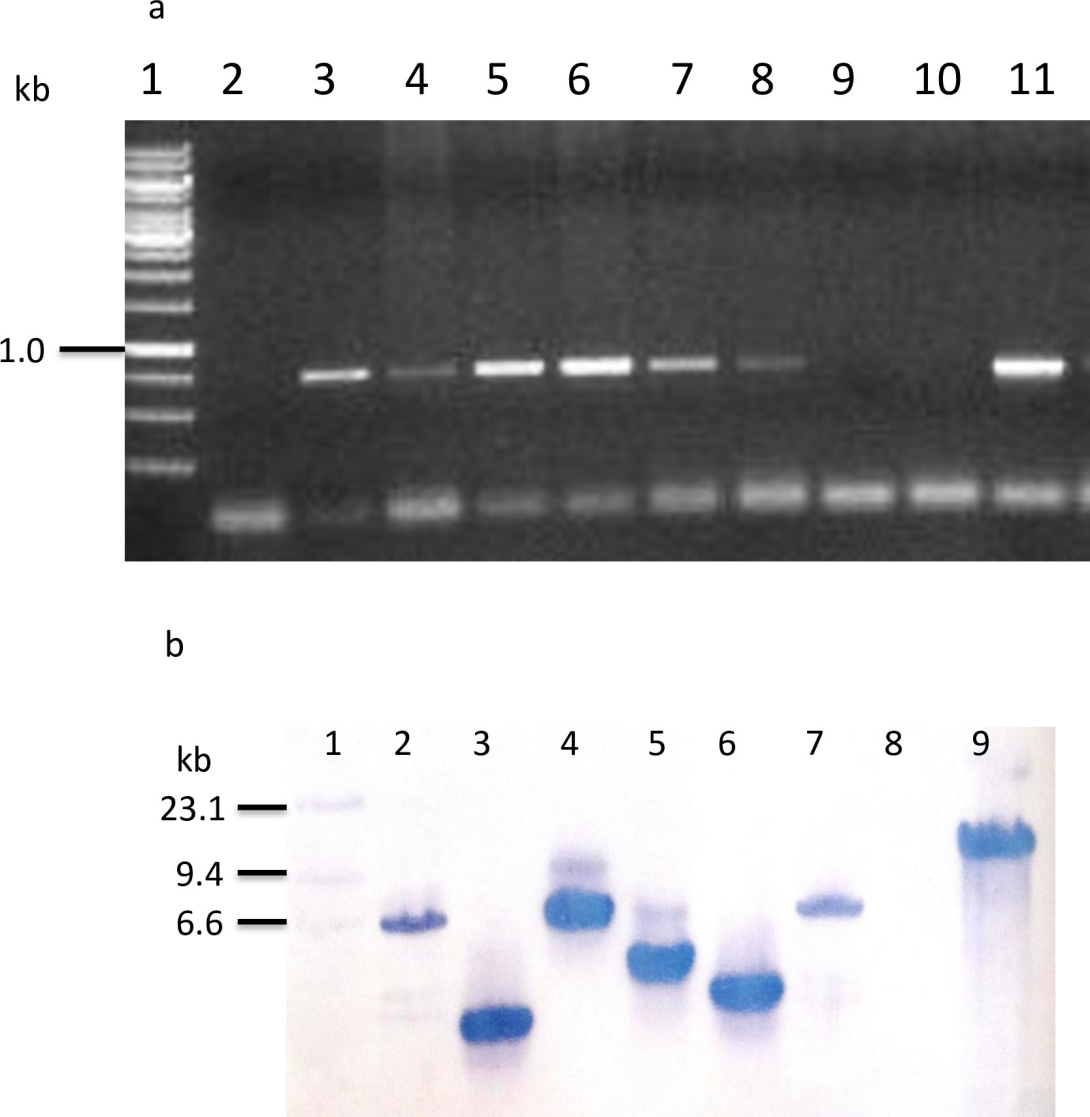
Presence of cm*Vip3Aa* in transgenic chickpea lines. (a) PCR amplification of cm*Vip3Aa* gene segment [L1: 1kb DNA ladder, L2: Control (DCP 92–3), L3: VPS 13, L4: VPS 14, L5: VPS 47, L6: VPS 57, L7: VPS 66, L8: VPS 77, L9: No template control (NTC), L10: Negative segregant of VPS 66, L11 Positive control (Recombinant Plasmid); (b) Genomic Southern blotting after single digestion (*Sal*I) [L1: DNA molecular weight marker II, DIG-labeled L2: VPS 13, L3: VPS 14, L4: VPS 47, L5: VPS 57, L6: VPS 66, L7: VPS 77, L8: Control (DCP 92–3), L9: Positive control (Recombinant Plasmid).

PCR analysis of the transgenic chickpea progenies in the T_1_ and T_2_ generation confirmed presence of expected 783 bp cm*Vip3Aa* specific amplification product, in progenies derived from five lines (Figs 2A and S3). Based on PCR screening of the T_1_ progenies derived from all primary transformants, progenies of 180 T_0_ lines were confirmed for the presence and transmission of cm*Vip3Aa*, with transformation efficiency of 4.30%. PCR analysis of T_1_ progenies derived from five lines indicated segregation pattern of transgene [VPS14: 8:3; VPS47: 4:0; VPS57: 3:0; VPS66: 3:0; VPS77:4:0], in accordance with copy number/locus (or loci) of integration (based on χ^2^ test with Yates’ correction) (S1 Table). Details of seeds harvested from all 180 T_0_ lines have been documented and submitted to Seed Repository (S2 Table). PCR analyses were also done to confirm transmission of cm*Vip3Aa* to T_2_ progenies derived from T_1_ positive chickpea plants. Based on PCR analyses, homozygous lines were identified (S3–S7 Tables; S4–S8 Figs) and selected for further molecular analysis and insect bioassay.

Southern blot hybridization of the selected six transgenic chickpea lines (T_2_ generation) indicated stable integration of cm*Vip3Aa* gene. The presence of single locus gene integration was detected in the transgenic lines *viz*. VPS13, VPS14, VPS66 and VPS77, whereas double loci in transgenic lines VPS47 and VPS57 (Figs 2B and S9). Genomic DNA digested with *Sal*I exhibited different banding pattern among the tested lines corresponding to integration locus(i) in the genome (*ca*. 6.5 kb, 4.1 kb and 3.9 kb for VPS13; 3.7 kb for VPS14; 9.4 kb and 6.5 kb for VPS47; 6.5 kb and 4.4 kb for VPS57; 4.1 kb for VPS66 and 6.6 kb for VPS77). Double digestion with *Nde*I and *Sal*I exhibited presence of 2.37 kb of cm*Vip3Aa* in the genome (S10 Fig), indicated intactness of inserted cassette in all six transgenic chickpea lines tested.

**Fig 3:**
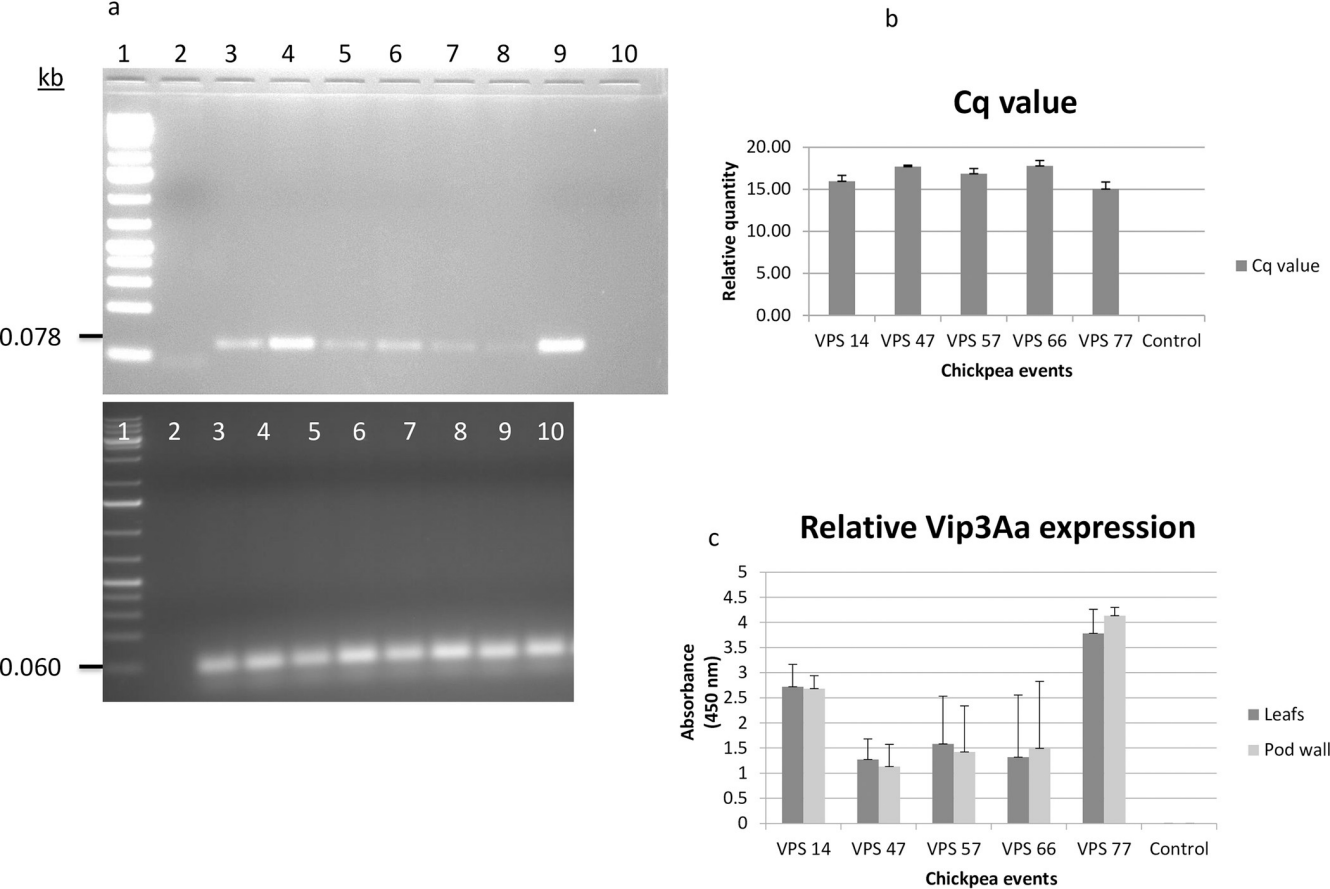
Expression of cm*Vip3Aa* in transgenic chickpea lines. (a) Panel 1: RT-PCR for cm*Vip3Aa* transcript (78 bp) in the transgenic chickpea lines [L1: 1 kb plus DNA ladder, L2: No Template Control L3& L4: VPS 14, L5: VPS 47, L6: VPS 57, L7: VPS 66, L8 and L9: VPS 77, L10: Control (DCP 92–3) Panel 2: RT-PCR for internal control, IF4α transcript (60 bp) in same lane order (b) Comparative expression of cm*Vip3Aa* in leaves of five transgenic lines and control (c) Absorbance (450 nm) variation depicting cmVIP3Aa expression in leaves and pod walls of five transgenic chickpea lines and control.

RT-PCR analysis of total transcripts indicated presence of cm*Vip3Aa* transcript (78 bp) in the transgenic lines tested, which was missing in the non-transgenic counterpart (Figs 3A, S11 and 12). Real-time PCR based quantitative estimation of cm*Vip3Aa* specific transcript indicated substantially higher levels of cm*Vip3Aa* in the tested transgenic lines, with average Cq values ranging from 15.01±0.86 to 19.32±0.10 (Fig 3B). The melting curve analysis indicated specificity of the amplified product generated during the real time PCR (S13 Fig).

Qualitative ELISA confirmed the expression of cmVIP3Aa protein in all the PCR positive chickpea progenies. In T_2_ generation, at post flowering stage a significant variation in the level of cmVIP3Aa expression were recorded in the leaves and pod wall. Using Bradford assay, total soluble protein was estimated as 6.09±0.07 ng/mg TSP to 10.95±0.08 ng/mg TSP (for leaf samples) and 5.34±0.02 ng/mg TSP to 9.95 ng/mg TSP (for pod wall samples). Absorbance (450 nm) corresponding to presence of cmVIP3Aa, as estimated by Qualitative ELISA kit varied from 0.2±0.08 to 4.12±0.03 (leaf samples) and 0.31±0.08 to 4.25±0.01 (pod walls), respectively, in all progenies derived from the five transgenic chickpea lines (Fig 3C), which was absent in non-transgenic line (*cv*. DCP 92–3).

### Insect bioassay

**Fig 4:**
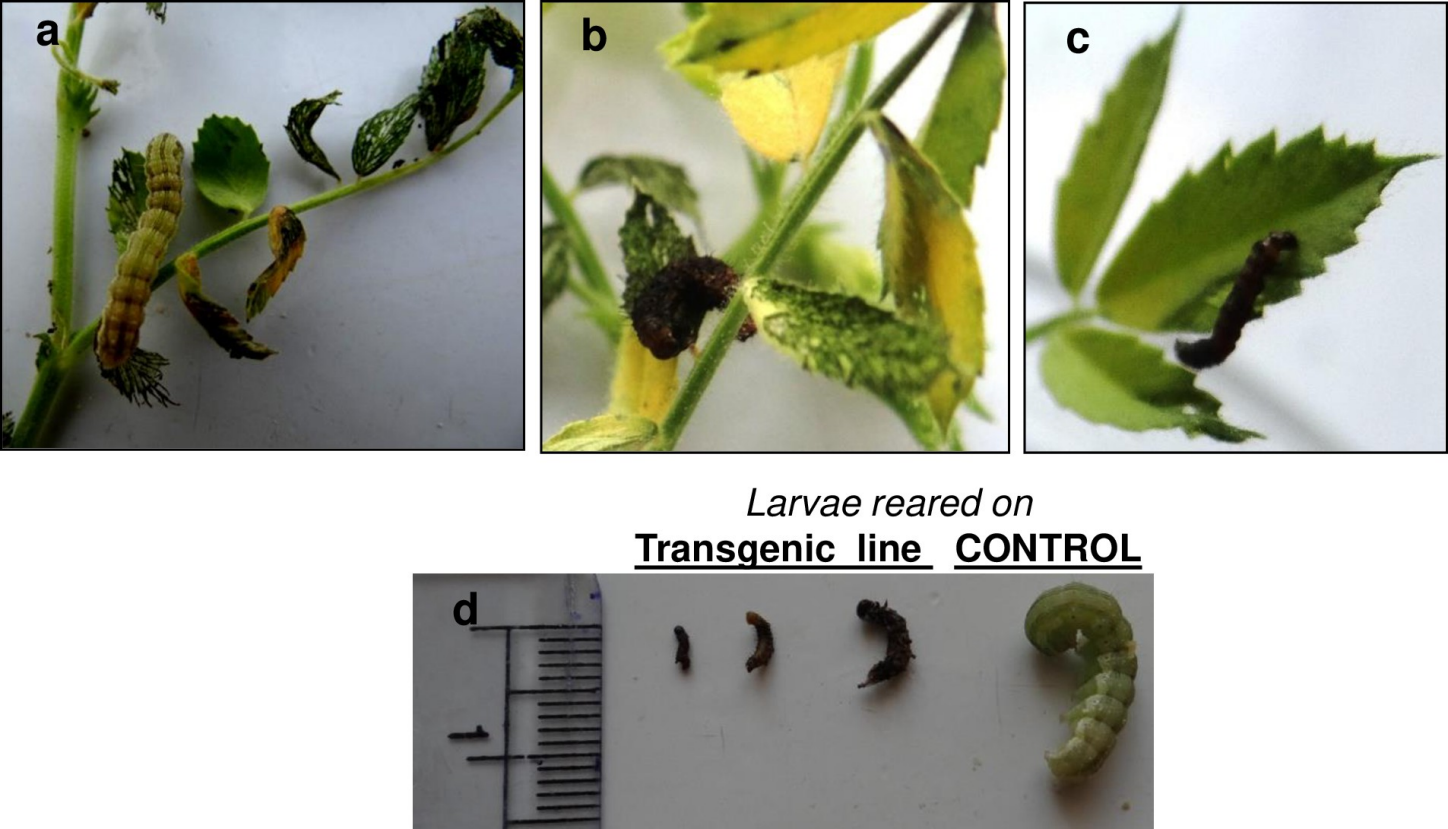
Insect Bioassay of transgenic chickpea lines against gram pod borer. (a) Pod borer larvae in control (DCP92-3) (b) Pod borer larvae in transgenic chickpea line (VPS 66) (c) Pod borer larvae in transgenic chickpea line (VPS 14) (d) Larval morphology, post no choice bioassay in transgenic line (dead larvae) and control.

Young chickpea leaves from T_2_ progenies derived from five transgenic chickpea lines VPS14, VPS47, VPS57, VPS66 and VPS77 were subjected to detached leaf insect (*H*. *armigera*) bioassay using 3^rd^ instar (5 days old) larvae. Significantly greater larval mortality was recorded in transgenic chickpea lines *viz*. VPS77 (39.75%) followed by VPS14 (27%) as compared to the control lines (DCP 92–3 (0%), P < 0.001) (Fig 4A–4C) (Table 1). No larval mortality was recorded in the control lines (DCP 92–3) in the experiments. Percentage reduction in leaf consumption per larva per plant over the control in transgenic chickpea lines ranged from 38.35 to 82.91% indicating efficacy of the transgenic lines by reduction in defoliation percentage. The highest mean percent reduction in leaf weight consumption over control was recorded from the transgenic line, VPS77 (82.91) followed by VPS57 (63.61) and VPS66 (60.05) which were higher than VPS14 (51.4) and VPS47 (38.35). Average percent reduction in larval weight gain over control was significantly higher on transgenic lines, VPS77 (68.23) followed by VPS57 (47.18), VPS14 (44.48), VPS66 (31.31) and VPS 47 (26.91). Further, retarded growth and development were exhibited in the larvae tested on transgenic lines as compared to control (Fig 4D). The regression analysis indicated co-relation (R^2^ = 0.997) between cmVIP3Aa-protein concentration (Absorbance at 450 nm) with the larval mortality, indicating the effectiveness of cmVIP3Aa protein in larval mortality and growth reduction (Table 2). Based on the larva morphology, characteristic growth inhibition in terms of larval weight gain was witnessed among the surviving larvae tested on transgenic chickpea lines. Furthermore, the larva tested on transgenic lines exhibited delayed pupation compared to the larvae tested on control lines.

**Table 1:** Efficacy of tested transgenic chickpea lines against *H*. *armigera*.

Lines	Number of plants tested	% Larval mortality over control	Average leaf weight consumed (mg)/ larvae/ plant	Average larvae weight gain (mg)/larvae/plant	% Reduction in leaf consumption over control	% Reduction in larval weight gain over control
VPS 14	5	27 (30.91^a,b^)	97.5	27.14	51.4 (45.84^b,c^)	44.48 (41.80^a,b^)
			90.14	21.71		
			51.67	10.33		
			84.2	26.8		
			70.4	20.6		
VPS 47	3	12.33 (16.97^b,c^)	92.3	27.7	38.35 (38.24^c^)	26.91 (31.25^b^)
			99.0	28.0		
			108.5	28.5		
VPS 57	3	14.67 (18.65^a,b,c^)	58.0	30.0	63.61 (53.63^a,b^)	47.18 (43.35^a,b^)
			96.2	22.22		
			30.0	8.67		
VPS 66	3	15.67 (19.01^a,b,c^)	70.0	27.57	60.05 (50.88^b,c^)	31.31 (33.65^b^)
			47.0	20.22		
			76.0	31.33		
VPS 77	4	39.75 (39.03^a^)	27.5	5.0	82.91 (65.81^a^)	68.23 (56.22^a^)
			30.43	11.00		
			56.67	16.0		
			31.67	17.0		
Control	4	0.00	163.2	42.7	-	-
			161.0	34.1		
			204.0	34.2		
			224.0	42.9		
CV	-	51.60	-	-	14.49	25.13

Values within parenthesis is *arc-sine* transformed values.

Mean followed by same letters are not significantly different from each other by DMRT (P<0.01).

**Table 2:** cmVIP3Aa specific absorbance (at 450 nm) *vis a vis* larval mortality in transgenic chickpea.

Events	% Larval mortality	Absorbance (450 nm)
VPS 14	27.00	2.7
VPS 47	12.33	1.2
VPS 57	14.67	1.5
VPS 66	15.67	1.405
VPS 77	39.75	3.96
Control	0	0

R^2^ = 0.997, P<0.01.

## Discussion

Chickpeas are protein-rich grain legumes crucial for nutritional security and sustainable agriculture. The gram pod borer (*Helicoverpa armigera* Hubner) is the most devastating insect pest of chickpea and till date, no complete-resistance source has been identified in chickpea gene pool. The pod borer exhibits *facultative diapause* that favors its survival in adverse weather conditions (winter and summer seasons) [38]. Amelioration of insect damage can add to chickpea production basket, reduce insectide use and help in sustainable production scenario augmenting nutritional security.

Insecticidal genes derived from the bacteria; *Bacillus thuringiensis* (*Bt*) are efficacious against major classes of insects. Use of single gene-resistance may not provide sustainable protection for longer period as observed in first generation (single gene) transgenic plants. Reports of insects overcoming resistance employing a number of mechanisms including reduced pro-toxin activation, rapid proteolytic degradation, attenuated binding of CRY toxins to the respective cognate receptors due to gene mutations, or reduced expression of corresponding receptors *via*. trans-regulation, are beginning to emerge. Besides, the epigenetic mechanisms, host intestinal microbiota and detoxification enzymes also play significant roles in the insects’ resistance against CRY toxins [39–41]. Several strategies have been adopted to manage resistance to Bt toxins, however, stacking or pyramiding toxin gene(s) with distinct mode of action and/or receptor binding site is a preferred technique [42].

Pyramiding *Vip* and *Cry* gene is considered a suitable strategy for resistance management, as VIP exhibit less structural and sequence homology with CRY proteins [43,44]. Transgenic cotton (VipCot^TM^) expressing both *Vip3A* and *Cry1Ab* was observed to be more effective in controlling lepidopteran pests compared to *Cry1Ac* singly [45,46]. Similar observation was witnessed in transgenic maize (Agrisure Viptera^R^ 3110) expressing a pyramided (*Vip3A* and C*ry1Ab*) gene cassette [47].

Earlier report suggested requirement of higher level of bacterial derived insecticidal proteins in host plant for effective resistance against the target pest, because of differential prokaryotic and eukaryotic codon usage or preference [48]. Factors like codon usage bias, AT-richness, presence of cryptic poly-adenylation signals and mRNA destabilizing sequence motifs tends to lower the expression of *Bt* genes in transgenic plant system [49]. To maximize *Vip3Aa* gene expression in transgenic chickpea cells, the coding sequence of the *Bt* derived native *Vip3Aa* gene was modified using the strategy described earlier [50]. Interestingly, in the current study, the GC content (30%) remained unaltered compared to native sequence, post-optimization. Crop based codon modification was earlier adopted for enhanced expression in cotton [51], soybean [52], chickpea [35], cowpea [53] and organellar expression [54].

Chickpeas are amenable to genetic manipulation (direct and indirect methods), with very low efficiency [55,56]. Particle gun-based methods have the potential to overcome genotype barrier/dependency to transformation, co-transform two or more genes (stacking/pyramiding) and clean gene technology to address biosafety issues [57]. In the present study we have successfully adapted a standardized protocol [34] for developing transgenic chickpea plants without using any plant selectable marker in the transformation process. This is a very laborious task; however, once lines are identified it ensures only presence of gene of interest in the progenies/lines. Further, we do not rule out the possibility of presence of bacterial selection marker (vector backbone) in the generated plants (data not shown). In the current study, the transmission of the transgene/inheritance was confirmed by PCR analysis at T_1_ and T_2_ stages and molecular analyses and transformation efficiency were calculated based on analyses thereof. The transmission and organization of the transgene were evaluated based on PCR analysis of five selected transgenic chickpea lines. Distorted segregation pattern were observed in several developed chickpea lines and further research is required to understand this mechanism. Mixed population of homozygous and hemizygous lines could also be identified at T_1_/T_2_ stages. Notably, homozygous lines appeared in very few instances, as per PCR analysis of T_2_ progenies derived from PCR positive T_1_ plants derived from all the events. The confirmed progenies were only selected and evaluated for characterization of the transgenic lines. Southern blot analyses of the generated transgenic lines indicate presence of single and multiple copies of the transgene. Earlier studies based on twin T-DNA strategy towards development of marker-free chickpea lines indicated that the efficiency of recovery of marker free lines is too low to be practical [3].

Direct transformation has been used to obtain fertile transgenic chickpea lines that have significant higher level of transient expression of reporter gene (GUS), comparable to earlier reports in soybean [52]. In the current study, transformation efficiency of 4.30% was achieved, which is comparable with the earlier reports [58,59] and higher than our earlier report on *Agrobacterium tumefaciens* mediated genetic transformation (0.076%) of same genotype of chickpea (*cv*. DCP 92–3) [35].

Estimation of protein of interest is an important aspect in characterization of transgenic lines. However, commercial quantitative ELISA kits are not available/accessible. We hypothesized that absorbance (450 nm) should correspond to protein concentration [60], as estimated by qualitative ELISA kit. However, further investigation shall provide better insights to understand protein concentration and mortality values of target insect. Variable levels of foreign protein expression have been reported for different plant tissues collected at different developmental stages in chickpea, based on promoters driving the gene expression and other associated regulatory elements [13,35]. In general, protein expression during pre-flowering vegetative stages was reported higher as compared to the post-flowering stage [13]. Hence, the major emphasis was enhancing the protection post flowering at target tissues like pod wall, using pod wall specific *msg* promoter [13]. In the current study, sustained expression level of cm*Vip3Aa* were observed in leaf and pod-wall tissues of transgenic chickpea lines, post flowering stage, where the chickpea plants were infested by larvae, besides terminal heat/drought stress in field conditions. This may possibly be due to the presence an alcohol dehydrogenase *(ADH)* gene-derived 5’-untranslated region (5’-UTR) downstream of the cauliflower mosaic virus (CaMV promoter) derived 35S promoter and presence of a heat shock protein *(HSP)* gene derived terminator that promotes/sustains the expression, as demonstrated earlier, [61–63] besides codon modification of the *Vip3Aa* gene.

Recently, Vip proteins are explored for engineering insect resistance in plants. Cowpeas expressing Vip3Ba are reported to cause *Maruca* larvae death [53]. In the contrary, Vip3A appears to be moderately toxic to *Helicoverpa* larvae, and the affect predominantly include growth abnormalities in the larvae at low toxin level. The variation is larval mortality data among the tested transgenic chickpea lines requires further research. Trait efficacy data presented here is at post-flowering stages with significant growth reduction of the insect larvae with minimal larval feeding (0.03–0.1g) recorded for the tested transgenic lines (0.18 g) compared to the control. Functional mortality (dead insect and those remaining at larval stages) better represents the effectiveness of Vip3Aa protein as reported earlier [64,65]. Moderate level of susceptibility of Vip3Aa against *Helicoverpa armigera* was also reported earlier. The quantitative analysis also indicated low susceptibility of *H*. *armigera* to the Vip3Aa toxins (with LC_50_ values 1660 ng/cm^2^). The term *functional mortality* has been adopted for precisely defining the toxicity of Vip3A class of toxins for growth inhibition and arresting larval development. Functional mortality in conjunction with other management strategies should be effective for enhanced resistance spectrum under field conditions [66]. In another study to explore the relationship between the structure and function, moderate level of toxicity with 47.22% insect (*H*. *armigera)* mortality was reported for native Vip3Aa protein, based on protein feeding assays [67]. Preliminary docking studies of native Vip3Aa protein with common APN and cadherin receptors, revealed conserved specific amino acid sites that govern insecticidal activity. Site-directed mutagenesis like Y619A in Vip3Aa could increase the resistance spectrum upto 75%. In another *in silico* study, Vip3Aa-Cry1Ac fusion protein was reported to exhibit strong affinity for *Lepidopteran* insect receptors [68]. Further research can expand our understanding of the resistance mechanism of Vip3Aa that can improve the insecticidal activity.

Here, we report synthesis of modified *Vip3Aa* gene encoding vegetative insecticidal protein (cmVIP3A), development of fertile, transgenic chickpea lines harboring cm*Vip*3Aa and their characterization. Single copy transgenic chickpea lines segregated as per Mendelian segregation pattern with transgene inheritance and expression to subsequent generations. The expression of the cmVIP3A protein, post-flowering stage provide moderate protection against gram pod borer with reduced larval feeding, and reduced larval weight gain, compared to control lines. This should help delay the resistance development in the insect under field conditions Discovery, validation and deployment of genes effective in controlling insect pest shall contribute in management of insect pest in chickpea.

## Supporting information

S1 Fig Confirmation of the recombinant plasmid [L1 and L6: 1kb DNA ladder, L2: Uncut plasmid, L3: Double digested (*Nde*I and SalI) plasmid, L4: Single digested (*Nde*I) plasmid, L5: Single digested (*Sal*I) plasmid]. (PDF)

S2 Fig Particle bombardment of chickpea and plant establishment. (a) Embryonic axis explants post bombardment (b) Explants with multiple shoots, (c) Elongation of shoots (d) Rooting of shoots,(e) Establishment of plantlet in matrix (f) Mature fertile chickpea plants.
(PDF)

S3 Fig Gel Image for PCR analysis (Fig 2A). (PDF)

S4 Fig Gel Image for PCR analysis of lines derived from VPS14.105 [L1: DNA Ladder, L2-L21: T2 progenies, L22: Control (DCP 92–3), L23: No Template Control (NTC); L24: Positive control (Recombinant Plasmid)]. (PDF)

S5 Fig Gel Image for PCR analysis of lines derived from VPS47.317 [L1: DNA Ladder, L2-L18: T2 progenies, L19: Control (DCP 92–3), L20: No Template Control (NTC); L21: Positive control (Recombinant Plasmid)]. (PDF)

S6 Fig Gel Image for PCR analysis of lines derived from VPS57.371 [L1: DNA Ladder, L2-L21: T2 progenies, L22: Control (DCP 92–3), L23: No Template Control (NTC); L24: Positive control (Recombinant Plasmid)]. (PDF)

S7 Fig Gel Image for PCR analysis of lines derived from VPS66.405 [L1: DNA Ladder, L2-L23: T2 progenies, L24: Control (DCP 92–3), L25: No Template Control (NTC); L26: Positive control (Recombinant Plasmid)]. (PDF)

S8 Fig Gel Image for PCR analysis of lines derived from VPS77.421 [L1: DNA Ladder, L2-L22: T2 progenies, L23: Control (DCP 92–3), L24: No Template Control (NTC); L25: Positive control (Recombinant Plasmid)]. (PDF)

S9 Fig Full length blots (Fig 2B). (PDF)

S10 Fig Full length blots of Genomic Southern blotting after double digestion (*Nde*I and *Sal*I) [L1: DNA molecular weight marker II, DIG-labeled, L2: VPS 13, L3: VPS 14, L4: VPS 47, L5: VPS 57, L6: VPS 66, L7: VPS 77, L8: Control (DCP 92–3), L9: Positive control (Recombinant Plasmid). (PDF)

S11 Fig Gel Image for RT-PCR analysis (Fig 3A-Upper Panel). (PDF)

S12 Fig Gel Image for RT-PCR analysis (Fig 3A-Lower Panel). (PDF)

S13 Fig Melt curve analysis of Real Time PCR experiment. (PDF)

S1 Table Chi-square test to understand segregation pattern of of *Vip3Aa* in T_1_ progenies of five independent chickpea lines. (PDF)

S2 Table Details of 180 T_0_ transgenic chickpea lines and seeds harvested. (PDF)

S3 Table Details of PCR analysis of lines derived from VPS14 (T_1_ and T_2_ stages). (PDF)

S4 Table Details of PCR analysis of lines derived from VPS47 (T_1_ and T_2_ stages). (PDF)

S5 Table Details of PCR analysis of lines derived from VPS57 (T_1_ and T_2_ stages). (PDF)

S6 Table Details of PCR analysis of lines derived from VPS66 (T_1_ and T_2_ stages). (PDF)

S7 Table Details of PCR analysis of lines derived from VPS77 (T_1_ and T_2_ stages). (PDF)

S1 Raw images (PDF)

S1 Info Complete coding sequence (CDS) of cm*Vip3Aa* and sequence alignment with native Vip3Aa. (PDF)

S2 Info Primary amino acid sequence of cmVIP3Aa protein and sequence alignment with native VIP3Aa. (PDF)
