# Supplementary figures and images for: Evaluation of transgenic chickpea harboring codon-modified Vip3Aa against gram pod borer (Helicoverpa armigera H.)
Source: PLoS One. 2022 Jun 24;17(6):e0270011. doi: 10.1371/journal.pone.0270011 (PMC9231776; doi:10.1371/journal.pone.0270011)

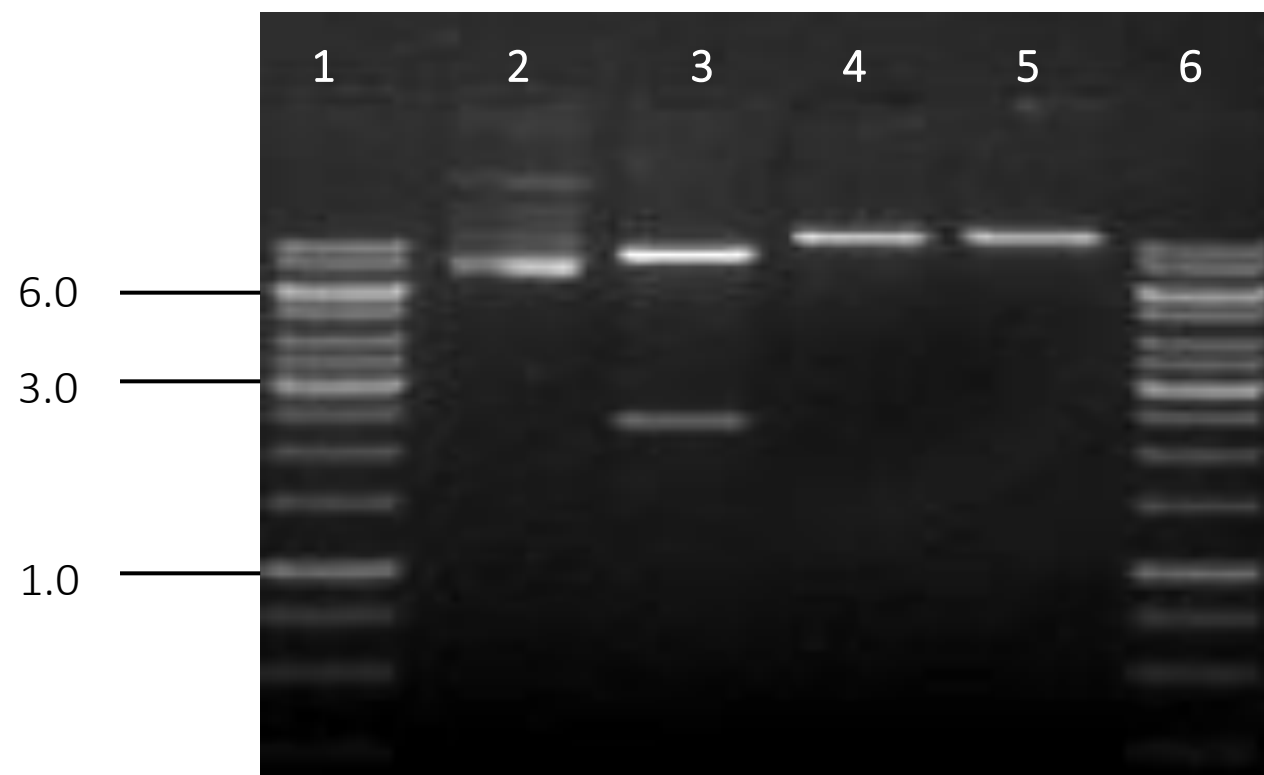

**S1 Fig**

Supplement: S1 Fig — (PDF) [file pone.0270011.s001.pdf]

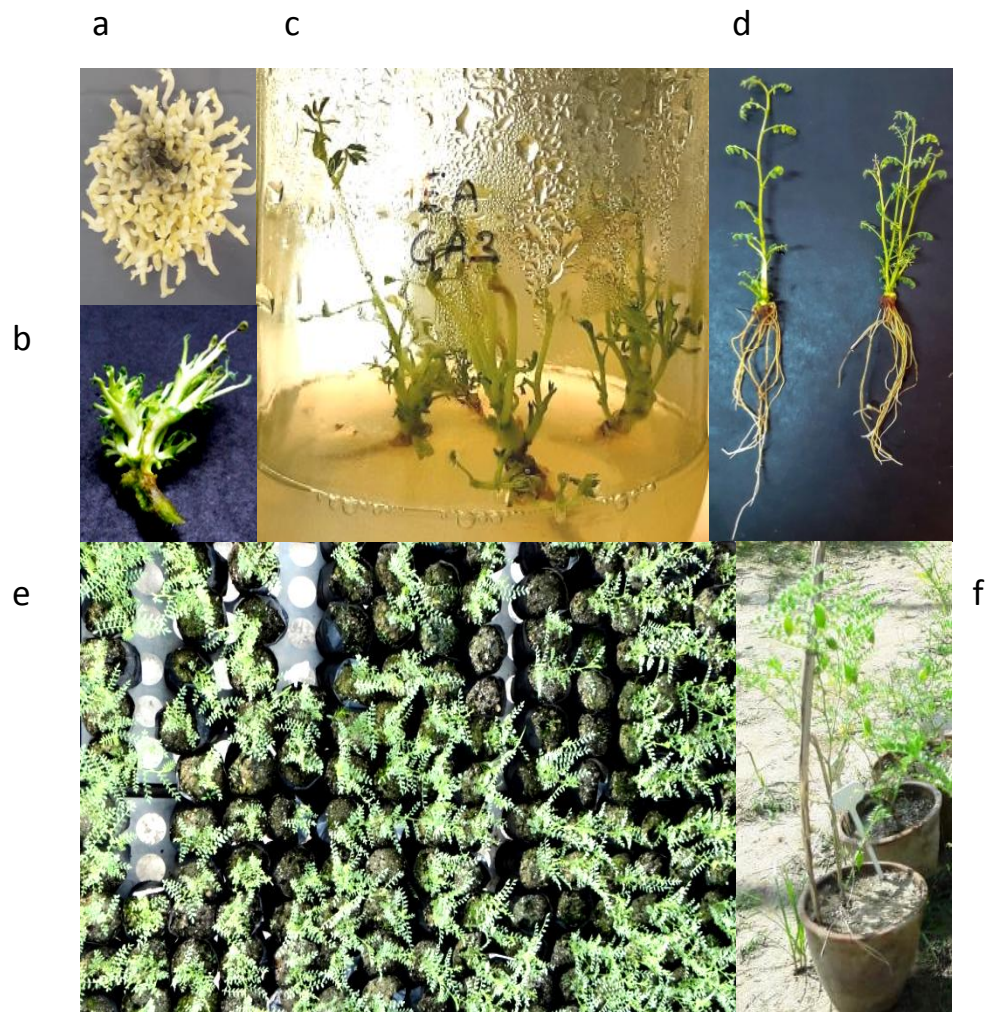

**S2 Fig**

Supplement: S2 Fig — (a) Embryonic axis explants post bombardment (b) Explants with multiple shoots, (c) Elongation of shoots (d) Rooting of shoots,(e) Establishment of plantlet in matrix (f) Mature fertile chickpea plants. (PDF) [file pone.0270011.s002.pdf]

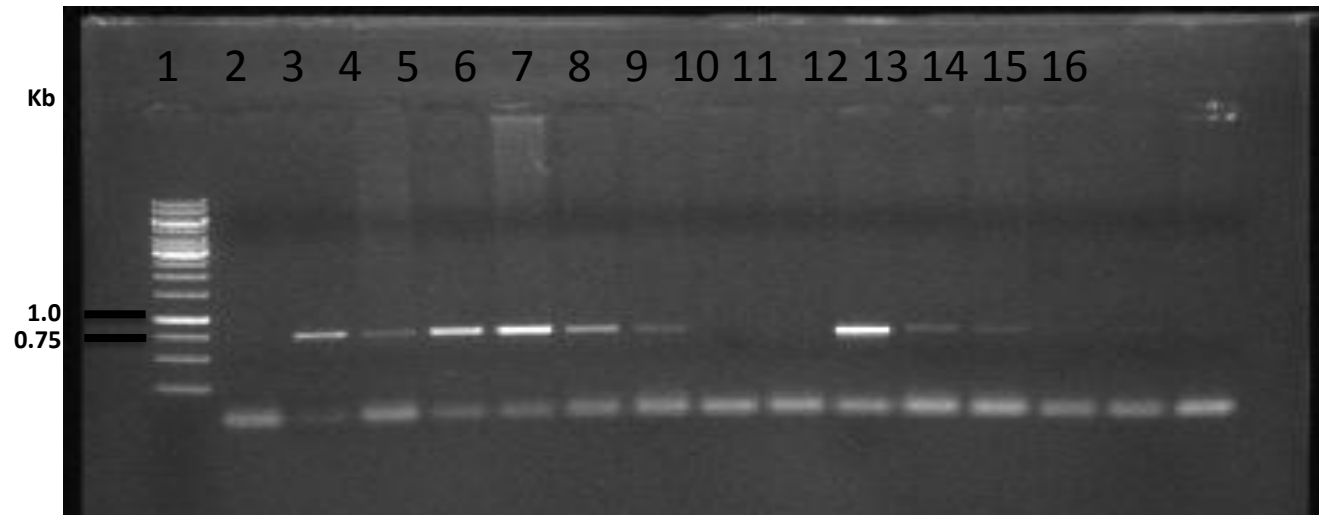

**S3 Fig**  
**(For Fig 2a)**

Supplement: S3 Fig — (PDF) [file pone.0270011.s003.pdf]

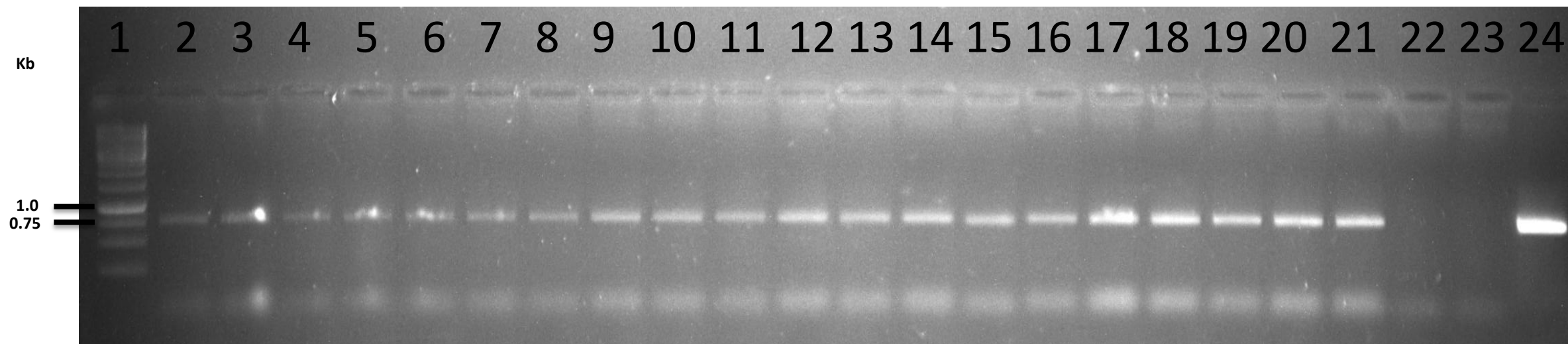

**S4 Fig**

Supplement: S4 Fig — (PDF) [file pone.0270011.s004.pdf]

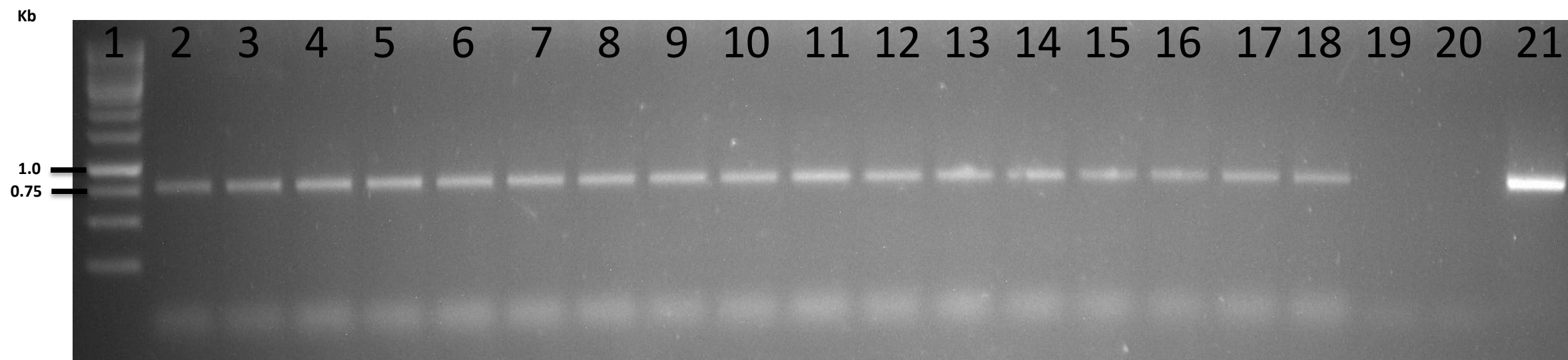

**S5 Fig**

Supplement: S5 Fig — (PDF) [file pone.0270011.s005.pdf]

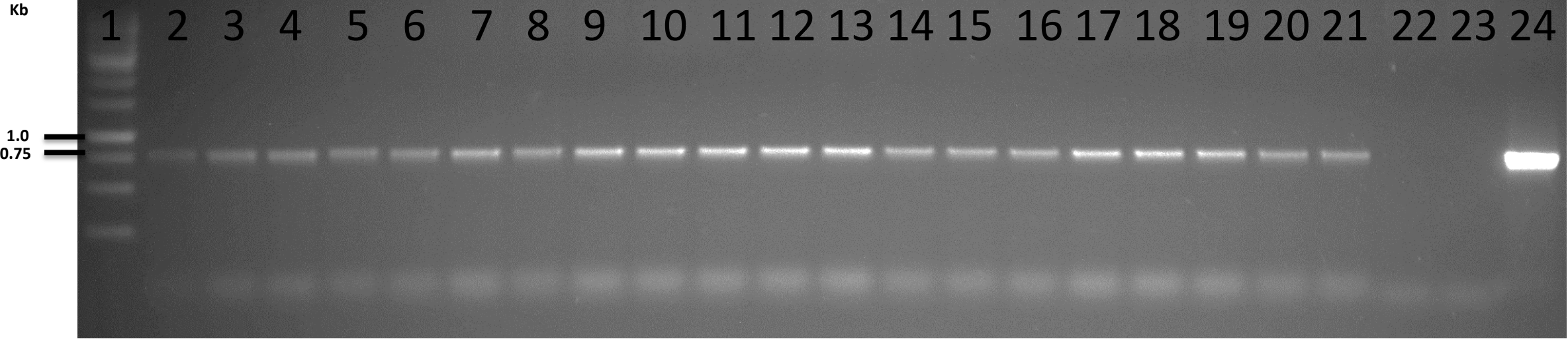

S6 Fig

Supplement: S6 Fig — (PDF) [file pone.0270011.s006.pdf]

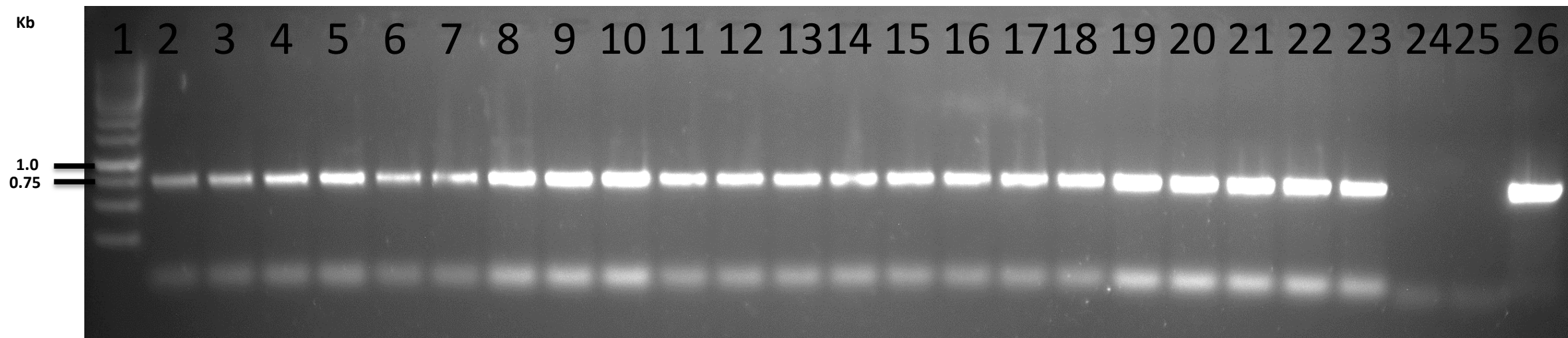

**S7 Fig**

Supplement: S7 Fig — (PDF) [file pone.0270011.s007.pdf]

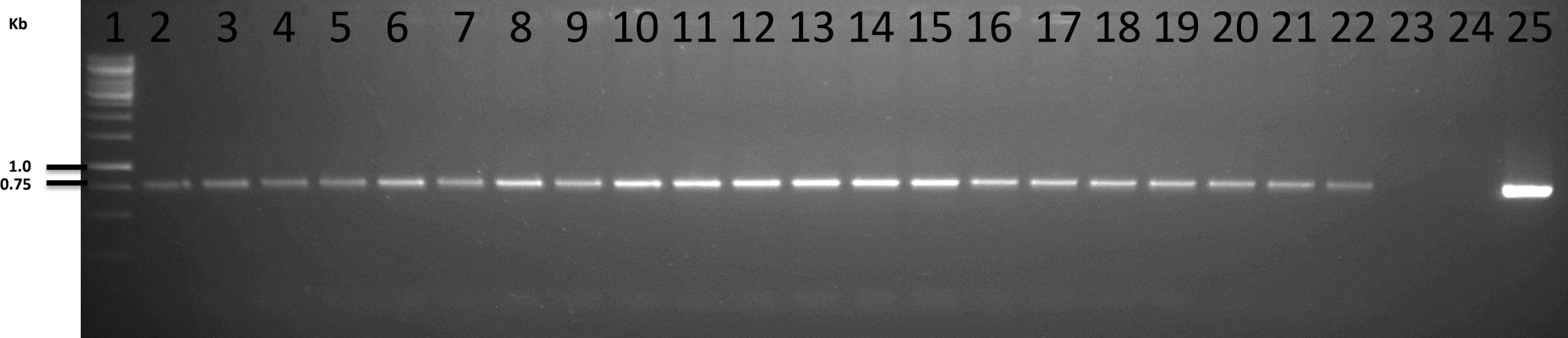

**S8 Fig**

Supplement: S8 Fig — (PDF) [file pone.0270011.s008.pdf]

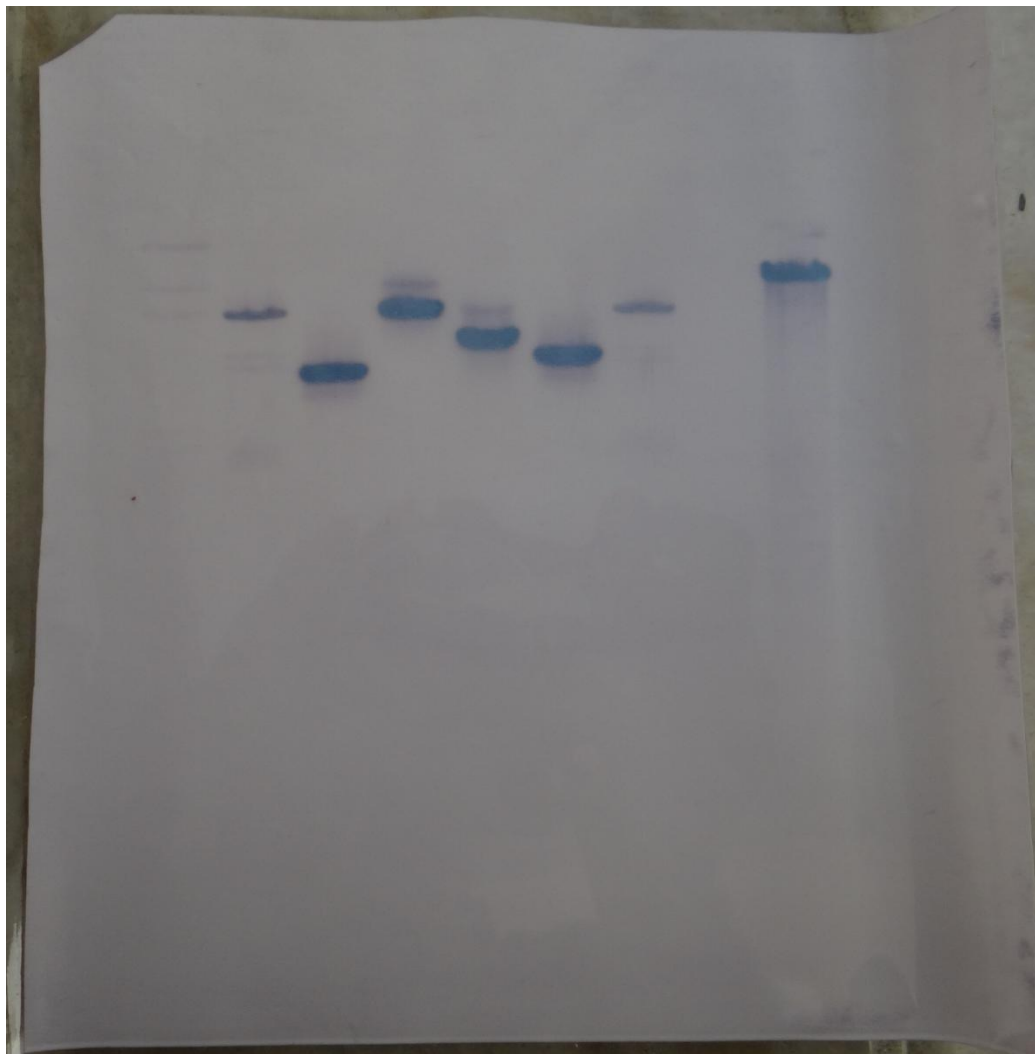

**S9 Fig**  
**(For Fig 2b)**

Supplement: S9 Fig — (PDF) [file pone.0270011.s009.pdf]

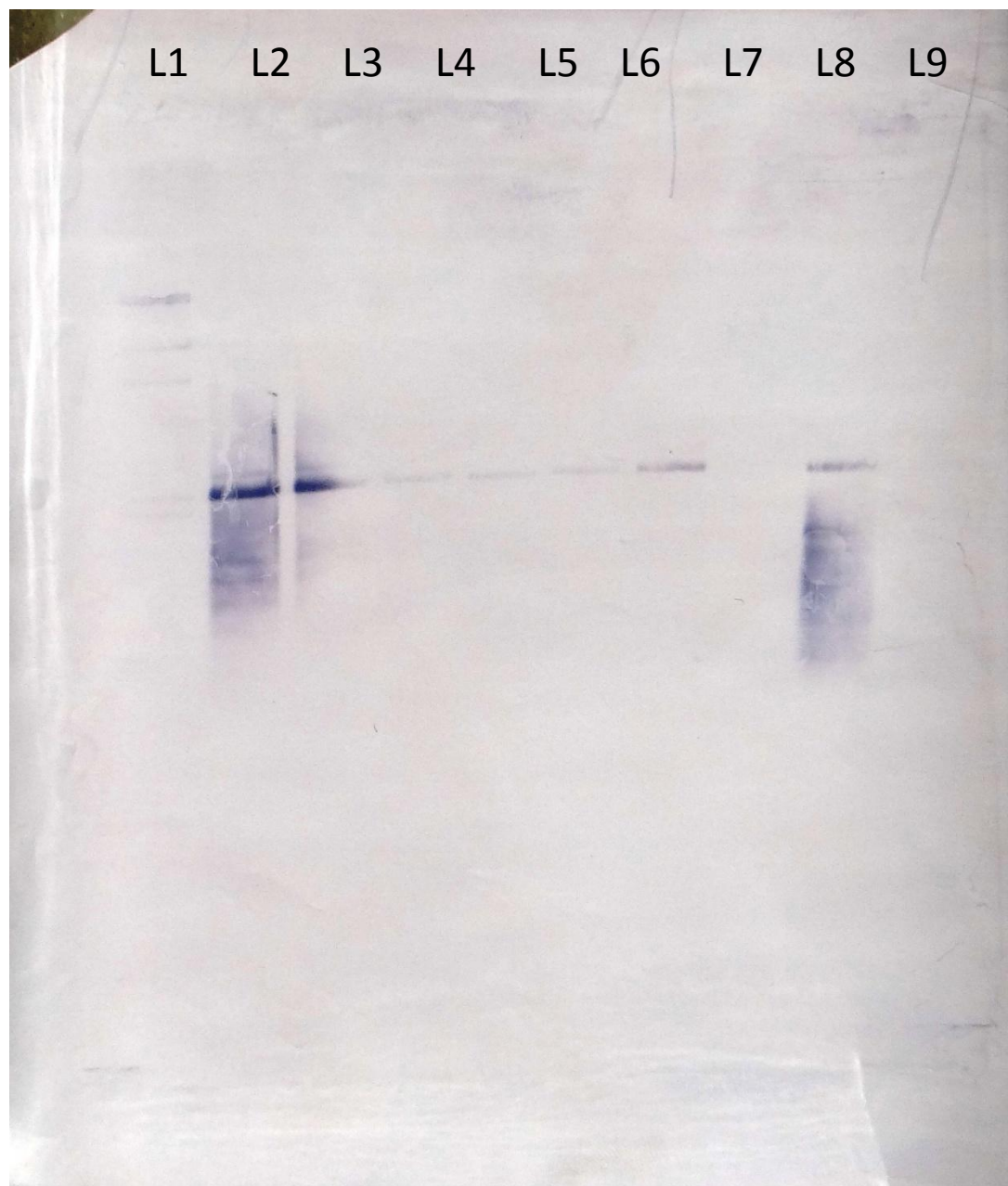

**S10 Fig**

Supplement: S10 Fig — (PDF) [file pone.0270011.s010.pdf]

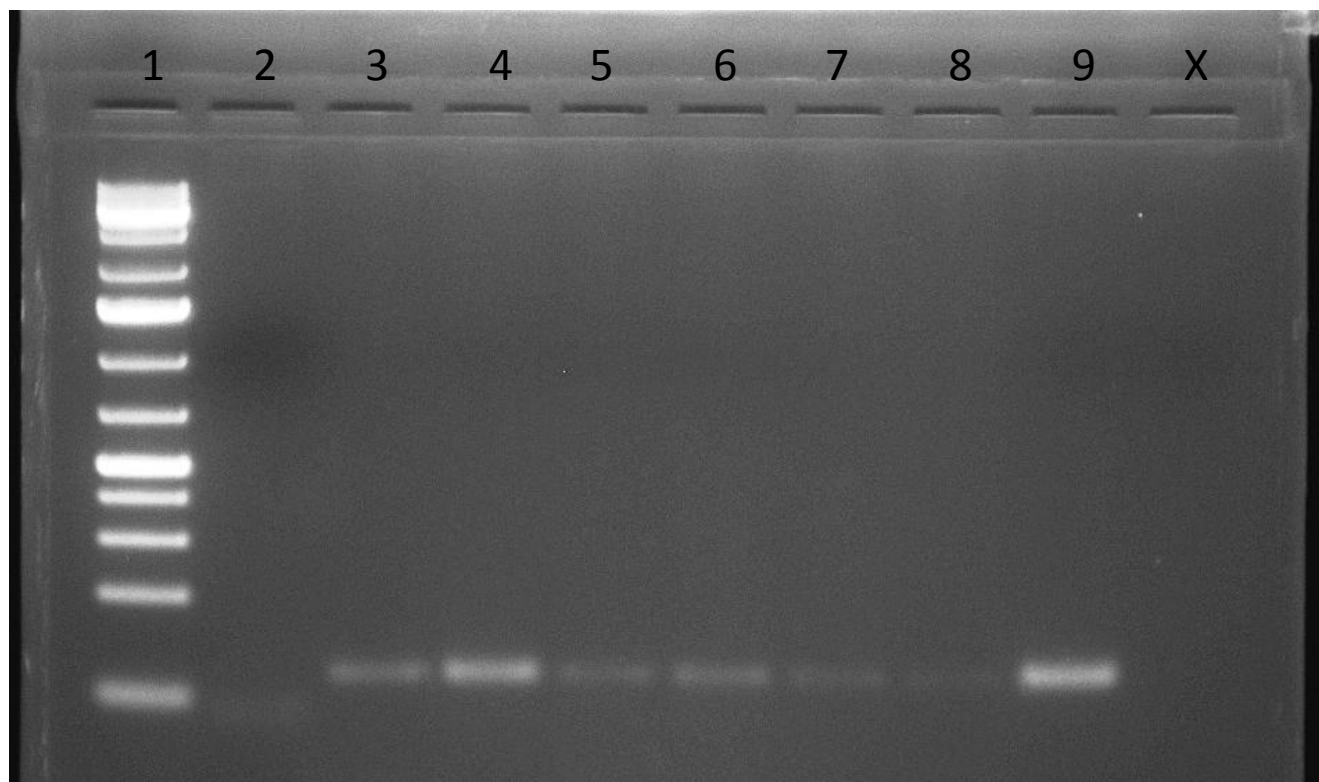

**S11 Fig**  
**(For Fig 3a Upper Panel)**

Supplement: S11 Fig — (PDF) [file pone.0270011.s011.pdf]

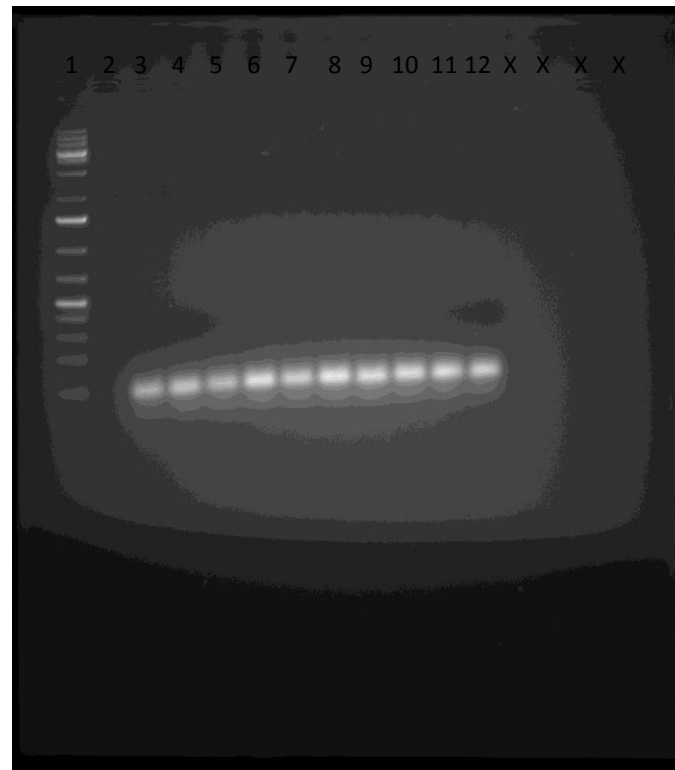

**S12 Fig**  
**(For Fig 3a Lower Panel)**

Supplement: S12 Fig — (PDF) [file pone.0270011.s012.pdf]
