# Supplementary material for: Evaluation of transgenic chickpea harboring codon-modified Vip3Aa against gram pod borer (Helicoverpa armigera H.)
Source: PLoS One. 2022 Jun 24;17(6):e0270011. doi: 10.1371/journal.pone.0270011 (PMC9231776; doi:10.1371/journal.pone.0270011)
Supplement: S13 Fig — (PDF) [file pone.0270011.s013.pdf]

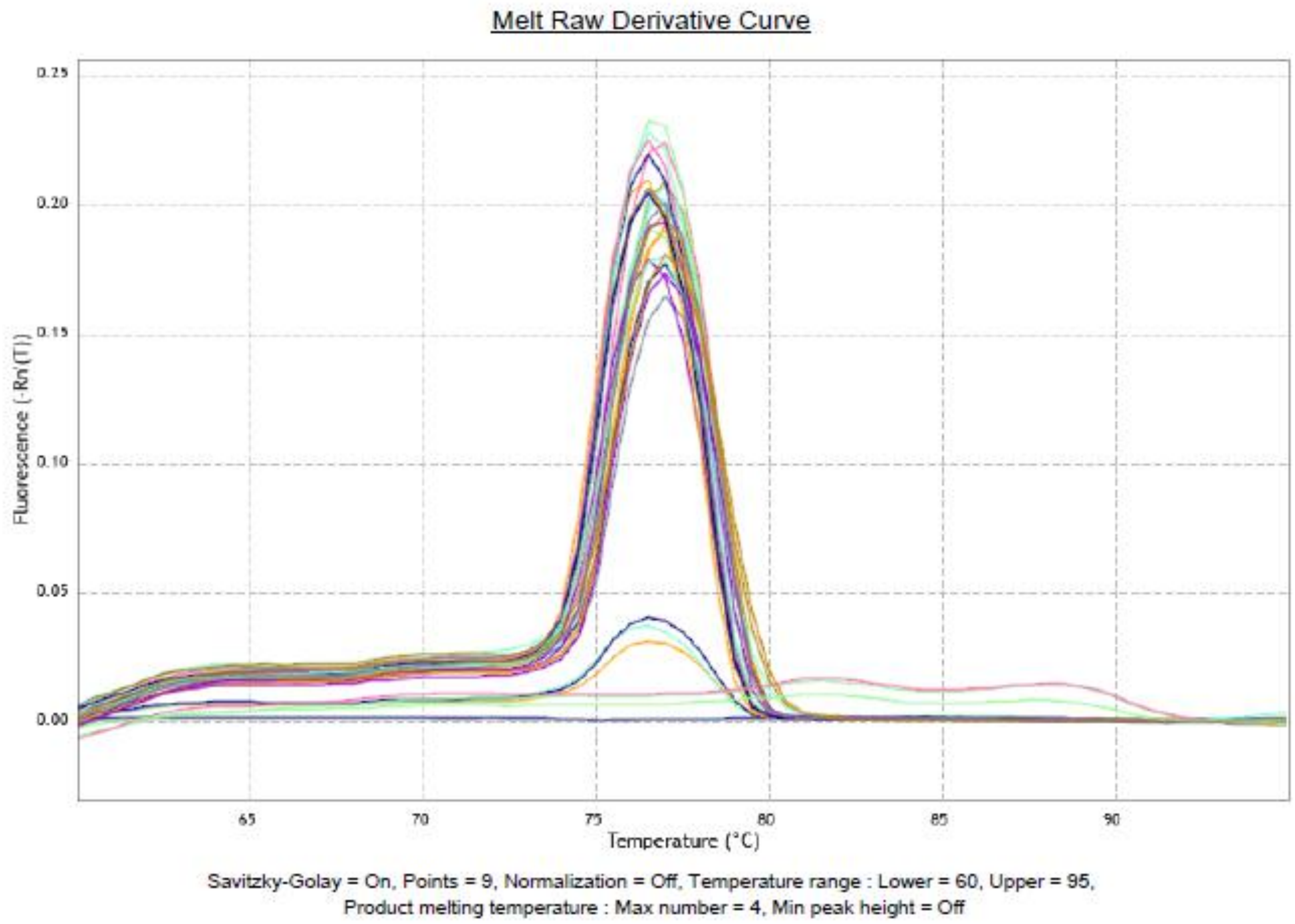
Melt Raw Derivative Curve
Fluorescence (-Rn'(T))
0.25
0.20
0.15
0.10
0.05
0.00
65
70
75
80
85
90
Temperature (°C)
Savitzky-Golay = On, Points = 9, Normalization = Off, Temperature range : Lower = 60, Upper = 95,
Product melting temperature : Max number = 4, Min peak height = Off

S13 Fig
