# Supplementary material for: Evaluation of transgenic chickpea harboring codon-modified Vip3Aa against gram pod borer (Helicoverpa armigera H.)
Source: PLoS One. 2022 Jun 24;17(6):e0270011. doi: 10.1371/journal.pone.0270011 (PMC9231776; doi:10.1371/journal.pone.0270011)
Supplement: S1 Table — (PDF) [file pone.0270011.s014.pdf]

S1 Table

T_0 lines	Total T_1 seeds harvested	Copy number*	PCR (+) progenies	PCR (-) progenies	Observed ratio	Chi-square value^
VPS 14	11	1	8	3	8:3	0.03
VPS 47	4	2	4	0	4:0	0.26
VPS 57	3	2	3	0	3:0	0.20
VPS 66	3	1	3	0	3:0	1.00
VPS 77	4	1	4	0	4:0	1.33

*Based on Southern Blot data

^Against 3:1 (Single copy insert) and 15:1 (Two copy/loci inserts) with Yates' correction (Critical chi square at 0.01; df: 1; Value = 2.71)
