# Supplementary material for: Evaluation of transgenic chickpea harboring codon-modified Vip3Aa against gram pod borer (Helicoverpa armigera H.)
Source: PLoS One. 2022 Jun 24;17(6):e0270011. doi: 10.1371/journal.pone.0270011 (PMC9231776; doi:10.1371/journal.pone.0270011)
Supplement: S2 Table — (PDF) [file pone.0270011.s015.pdf]

S2 Table

S. No.	T_0 Lines	PCR Analysis of T_1 transgenic progenies		PCR positive T_1 plant codes	No. of T_2 seeds for PCR positive lines
		Presence of Vip3Aa	Absence of Vip3Aa		
1	VPS 1	3	9	6,7, 8	46
2	VPS 2	1	10	22	14
3	VPS 5	2	4	42, 43	52
4	VPS 7	1	2	60	27
5	VPS 8	1	5	66	16
6	VPS 12	3	5	84, 85, 86	96
7	VPS 13	4	0	87, 89, 90, 91	69
8	**VPS 14**	**8**	**3**	**92, 93, 94, 95,102,103, 104, 105**	**177**
9	VPS 15	1	2	109	18
10	VPS 16	3	4	110, 112,116	84
11	VPS 17	2	5	122, 123	61
12	VPS 18	3	4	125, 129, 131	76
13	VPS 19	4	2	132, 133, 134, 136	96
14	VPS 20	3	5	139, 141, 142	84
15	VPS 21	5	4	143, 145, 146, 147, 150	125
16	VPS 22	3	4	154, 155, 158	98
17	VPS 23	1	2	160	16
18	VPS 24	2	1	163, 164	27
19	VPS 25	2	0	166, 167	33
20	VPS 26	2	5	170, 174	58
21	VPS 27	3	7	176, 180, 184	74
22	VPS 28	3	6	185, 190, 193	69
23	VPS 29	7	4	194, 195, 196, 198, 201, 202, 203	224
24	VPS 30	3	2	204, 206, 208	45
25	VPS 31	6	6	209, 211, 212, 214, 216, 220	94
26	VPS 36	0	2	-	-
27	VPS 37	3	6	226, 228, 231	62
28	VPS 38	4	2	234, 235, 236,239	84
29	VPS 39	3	5	240, 245, 246	59
30	VPS 40	6	3	248, 251, 252, 253, 254, 257	145

31	VPS 41	5	4	258, 260, 261, 262, 265	96
32	VPS 42	4	8	267, 268, 276, 279	84
33	VPS 43	8	6	280, 281, 282, 283, 284, 285, 286, 291	286
34	VPS 44	5	7	293, 295, 299, 300, 301	94
35	VPS 45	3	2	305, 308, 309	84
36	VPS 46	2	3	310, 313	60
37	**VPS 47**	**4**	**0**	**315, 316, 317, 318**	**113**
38	VPS 48	10	5	319, 320, 321, 322, 323, 324, 325, 326, 327, 331	311
39	VPS 50	5	3	332, 334, 335, 338, 339	145
40	VPS 51	4	2	341, 342, 344, 345	86
41	VPS 52	3	2	347, 348, 350	56
42	VPS 53	3	1	351, 352, 354	64
43	VPS 55	4	1	355, 356, 358, 359	91
44	VPS 56	2	1	366, 367	72
45	**VPS 57**	**3**	**0**	**368, 369, 371**	**83**
46	VPS 58	2	0	375, 378	43
47	VPS 59	1	0	382	37
48	VPS 60	1	0	385	17
49	VPS 61	3	2	393, 394, 395	74
50	VPS 62	3	1	396, 397, 398	99
51	VPS 64	2	0	401, 402	59
52	**VPS 66**	**3**	**0**	**403, 404, 405**	**60**
53	VPS 67	1	0	407	29
54	VPS 68	2	3	409, 410	65
55	VPS 69	1	2	411	27
56	VPS 70	2	3	412, 413	27
57	VPS 71	4	2	413, 414, 416, 417	68
58	VPS 72	1	2	420	29
59	**VPS 77**	**4**	**0**	**421, 422, 423, 424**	**106**
60	VPS 78	2	3	425, 430	36
61	VPS 79	5	3	431, 432, 435, 437,442	74
62	VPS 80	1	0	443	28
63	VPS 81	1	0	446	39

64	VPS 82	2	0	448, 450	32
65	VPS 83	4	3	452, 455, 463, 465	91
66	VPS 88	3	3	466, 469, 475	63
67	VPS 89	3	6	478, 486, 490	76
68	VPS 90	1	2	493	21
69	VPS 91	5	4	496, 500, 502, 504, 509	164
70	VPS 92	4	2	512, 515, 519, 522	84
71	VPS 93	2	0	523, 525	26
72	VPS 94	1	3	530	32
73	VPS 96	3	5	532, 536, 538	-
74	VPS 98	3	4	540, 543, 547	-
75	VPS 99	3	2	549, 550, 554	-
76	VPS 100	1	2	555, 558	-
77	VPS 101	3	3	559, 566	-
78	VPS 102	5	2	571, 572, 576, 578, 601	-
79	VPS 104	3	1	602, 603, 604	-
80	VPS 106	2	1	615, 616	-
81	VPS 107	1	0	618	-
82	VPS 108	3	2	621, 623, 624	-
83	VPS 110	3	2	628, 630, 631	-
84	VPS 111	1	0	632	-
85	VPS 112	1	0	633	-
86	VPS 113	2	4	637, 639	-
87	VPS 114	1	5	640	-
88	VPS 115	5	5	642, 645, 646, 648, 672	-
89	VPS 116	2	4	673, 679	-
90	VPS 118	3	6	682, 685, 688	-
91	VPS 119	4	3	692, 693, 694, 698	-
92	VPS 120	2		702, 704	-
93	VPS 121	3	2	711, 712, 714	-
94	VPS 122	4	1	720, 721, 723, 724	-
95	VPS 123	2	0	726, 727	-
96	VPS 124	2	3	731, 733	-
97	VPS 125	1	0	734	-
98	VPS 126	4	2	737, 738, 740, 741	-
99	VPS 128	3	2	746, 747, 748	-
100	VPS 130	3	2	750, 751, 753	-
101	VPS 131	2	2	755,756	-
102	VPS 133	3	1	760, 762, 763	-
103	VPS 134	4	1	765, 766, 768, 770	-
104	VPS 135	3	1	771, 773, 774	-

105	VPS 136	2	0	777, 778	-
106	VPS 137	3	2	779, 780, 783	-
107	VPS 139	1	0	786	-
108	VPS 140	2	3	787, 790	-
109	VPS 141	4	2	792, 793, 794, 796	-
110	VPS 142	2	4	799, 800	-
111	VPS 143	3	1	801, 803, 804	-
112	VPS 144	3	3	806, 808, 810	-
113	VPS 147	3	5	817, 819, 820	-
114	VPS 148	3	3	826, 827,831	-
115	VPS 149	1	0	832	-
116	VPS 150	2	3	834, 836	-
117	VPS 151	2	5	837, 838	-
118	VPS 153	4	3	843, 845, 846, 848	-
119	VPS 154	2	1	850, 852	-
120	VPS 155	2	0	853, 869	-
121	VPS 158	1	3	875	-
122	VPS 159	3	3	879, 882, 886	-
123	VPS 160	2	3	889, 893	-
124	VPS 161	2	1	895, 900	-
125	VPS 163	2	1	902, 904	-
126	VPS 164	2	3	906, 909	-
127	VPS 165	1	2	915	-
128	VPS 166	2	2	920, 921	-
129	VPS 167	2	1	925, 927	-
130	VPS 171	3	1	935, 940, 941	-
131	VPS 172	2	0	942, 946	-
132	VPS 175	1	4	949	-
133	VPS 176	1	2	952	-
134	VPS 179	1	2	958	-
135	VPS 180	1	1	962	-
136	VPS 184	1	1	971	-
137	VPS 186	2	0	975	-
138	VPS 187	2	2	979	-
139	VPS 189	1	3	983	-
140	VPS 192	1	0	985	-
141	VPS 193	2	0	986, 988	-
142	VPS 194	1	3	989	-
143	VPS 196	1	2	992	-
144	VPS 199	2	0	994, 995	-
145	VPS 200	1	2	997	-
146	VPS 201	2	1	1001, 1003	-
147	VPS 203	1	1	1005	-
148	VPS 205	2	0	1008, 1011	-
149	VPS 206	2	0	1015, 1019	-
150	VPS 207	1	0	1021	-
151	VPS 209	1	0	1023	-
152	VPS 210	1	2	1024	-

153	VPS 212	2	3	1032, 1033	-
154	VPS 213	1	3	1039	-
155	VPS 215	2	2	1042, 1044	-
156	VPS 217	3	1	1052, 1053, 1054	-
157	VPS 218	1	0	1057	-
158	VPS 219	2	3	1058, 1064	-
159	VPS 222	1	0	1068	-
160	VPS 223	2	0	1071, 1072	-
161	VPS 225	1	2	1077	-
162	VPS 226	3	1	1080, 1082, 1083	-
163	VPS 228	2	3	1085, 1088	-
164	VPS 229	1	0	1093	-
165	VPS 231	2	1	1096, 1099	-
166	VPS 235	2	1	1102, 1105	-
167	VPS 240	2	0	1115, 1117	-
168	VPS 241	1	0	1120	-
169	VPS 249	2	0	1133	-
170	VPS 252	1	3	1136	-
171	VPS 253	1	2	1139	-
172	VPS 283	1	2	1142	-
173	VPS 284	1	2	1145	-
174	VPS 377	1	3	1162	-
175	VPS 399	1	0	1178	-
176	VPS 405	1	0	1182	-
177	VPS 407	2	0	1186, 1187	-
178	VPS 411	3	1	1191, 1193, 1197	-
179	VPS 419	2	0	1201, 1203	-
180	VPS 420	2	0	1204, 1205	-

Note: "-" indicates not analysed
