# Supplementary material for: Evaluation of transgenic chickpea harboring codon-modified Vip3Aa against gram pod borer (Helicoverpa armigera H.)
Source: PLoS One. 2022 Jun 24;17(6):e0270011. doi: 10.1371/journal.pone.0270011 (PMC9231776; doi:10.1371/journal.pone.0270011)
Supplement: S3 Table — (PDF) [file pone.0270011.s016.pdf]

S3 Table

Event (T0)	T1				T2		
	Seeds	PCR (+)	PCR (-)	(Plant Code)	Seeds	PCR (+)	PCR (-)
VPS14	11	8	3	14.92	21	15	6
				14.93	31	20	11
				14.94	23	20	3
				14.95	25	17	8
				14.102	26	18	8
				14.103	09	9	0
				14.104	22	13	9
				14.105*	20	20	0
Total Seeds	11				177		

*Lines tested for bioassay
