# Supplementary material for: Evaluation of transgenic chickpea harboring codon-modified Vip3Aa against gram pod borer (Helicoverpa armigera H.)
Source: PLoS One. 2022 Jun 24;17(6):e0270011. doi: 10.1371/journal.pone.0270011 (PMC9231776; doi:10.1371/journal.pone.0270011)
Supplement: S4 Table — (PDF) [file pone.0270011.s017.pdf]

S4 Table

Event (T0)	T1				T2		
	Seeds	PCR (+)	PCR (-)	(Plant Code)	Seeds	PCR (+)	PCR (-)
VPS47	4	4	0	47.315	29	17	12
				47.316	36	20	16
				47.317*	17	17	0
				47.318	31	19	12
Total Seeds	4				113		

*Lines tested for bioassay
