# Supplementary material for: Evaluation of transgenic chickpea harboring codon-modified Vip3Aa against gram pod borer (Helicoverpa armigera H.)
Source: PLoS One. 2022 Jun 24;17(6):e0270011. doi: 10.1371/journal.pone.0270011 (PMC9231776; doi:10.1371/journal.pone.0270011)
Supplement: S5 Table — (PDF) [file pone.0270011.s018.pdf]

S5 Table

Event (T0)	T1				T2		
	Seeds	PCR (+)	PCR (-)	(Plant Code)	Seeds	PCR (+)	PCR (-)
VPS57	3	3	0	57.368	30	17	13
				57.369	33	21	12
				57.371*	20	20	0
Total Seeds	3				83		

*Lines tested for bioassay
