# Supplementary material for: Evaluation of transgenic chickpea harboring codon-modified Vip3Aa against gram pod borer (Helicoverpa armigera H.)
Source: PLoS One. 2022 Jun 24;17(6):e0270011. doi: 10.1371/journal.pone.0270011 (PMC9231776; doi:10.1371/journal.pone.0270011)
Supplement: S6 Table — (PDF) [file pone.0270011.s019.pdf]

S6 Table

Event (T0)	T1				T2		
	Seeds	PCR (+)	PCR (-)	(Plant Code)	Seeds	PCR (+)	PCR (-)
VPS66	3	3	0	66.403	21	15	6
				66.404	17	10	7
				66.405*	22	22	0
Total Seeds	3				60		

*Lines tested for bioassay
