# Supplementary material for: Evaluation of transgenic chickpea harboring codon-modified Vip3Aa against gram pod borer (Helicoverpa armigera H.)
Source: PLoS One. 2022 Jun 24;17(6):e0270011. doi: 10.1371/journal.pone.0270011 (PMC9231776; doi:10.1371/journal.pone.0270011)
Supplement: S7 Table — (PDF) [file pone.0270011.s020.pdf]

S7 Table

Event (T0)	T1				T2		
	Seeds	PCR (+)	PCR (-)	(Plant Code)	Seeds	PCR (+)	PCR (-)
VPS77	4	4	0	77.421*	21	21	0
				77.422	32	19	13
				77.423	17	7	10
				77.424	36	23	13
Total Seeds	4				106		

*Lines tested for bioassay
