# Supplementary material for: Evaluation of transgenic chickpea harboring codon-modified Vip3Aa against gram pod borer (Helicoverpa armigera H.)
Source: PLoS One. 2022 Jun 24;17(6):e0270011. doi: 10.1371/journal.pone.0270011 (PMC9231776; doi:10.1371/journal.pone.0270011)

Raw Figure 1: Confirmation of the recombinant plasmid [L1 and L6: 1kb DNA ladder, L2: Uncut plasmid, L3: Double digested (NdeI and SalI) plasmid, L4: Single digested (NdeI) plasmid, L5: Single digested (SalI) plasmid]

Raw Figure 2: Presence of cm*Vip3Aa* in transgenic chickpea lines

(a) PCR amplification of cm*Vip3Aa* gene segment [L1: 1kb DNA ladder, L2: Control (DCP 92-3), L3: VPS 13, L4: VPS 14, L5: VPS 47, L6: VPS 57, L7: VPS 66, L8: VPS 77, L9: No template control (NTC), L10: Negative segregant of VPS 66, L11 Positive control (Recombinant Plasmid);

(b) Genomic Southern blotting after single digestion (*Sal*I) [L1: DNA molecular weight marker II, DIG-labeled L2: VPS 13, L3: VPS 14, L4: VPS 47, L5: VPS 57, L6: VPS 66, L7: VPS 77, L8: Control (DCP 92-3), L9: Positive control (Recombinant Plasmid)

Raw Figure 3: Expression of cm*Vip3Aa* in transgenic chickpea lines

(a) RT-PCR for cm*Vip3Aa* transcript (78 bp) in the transgenic chickpea lines [L1: 1 kb plus DNA ladder, L2: No Template Control L3& L4: VPS 14, L5: VPS 47, L6: VPS 57, L7: VPS 66, L8 and L9: VPS 77, L10: Control (DCP 92-3**)**

(b) RT-PCR for internal control**,** IF4α transcript (60 bp) in same lane order, as above

Raw Figure 5: Full length blots of Genomic Southern blotting after double digestion (*Nde*I and *Sal*I) [L1: DNA molecular weight marker II, DIG-labeled, L2: VPS 13, L3: VPS 14, L4: VPS 47, L5: VPS 57, L6: VPS 66, L7: VPS 77, L8: Control (DCP 92-3), L9: Positive control (Recombinant Plasmid)

Raw Fig 1

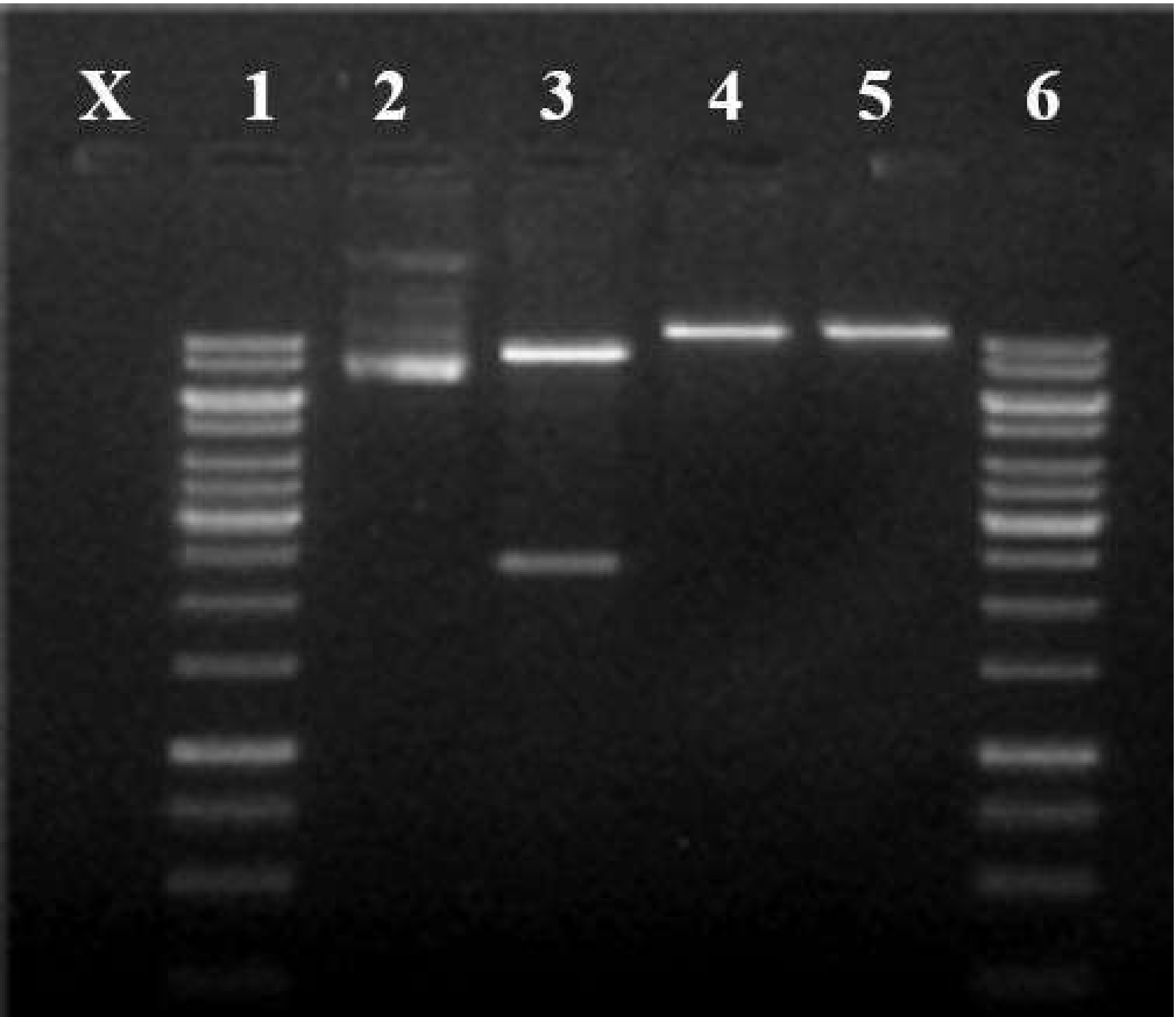

(a)Loading order: 1, 2, 3, 4, 5, 6 (L to R)
(b)Experimental samples: 1 to 6
(c)Instrument/Method used to capture the image: GelDoc XR (BioRad, USA)
(d) Image code in the main manuscript: Figure not part of the main manuscript, available as Supplementary figure 1

Raw Fig 2a

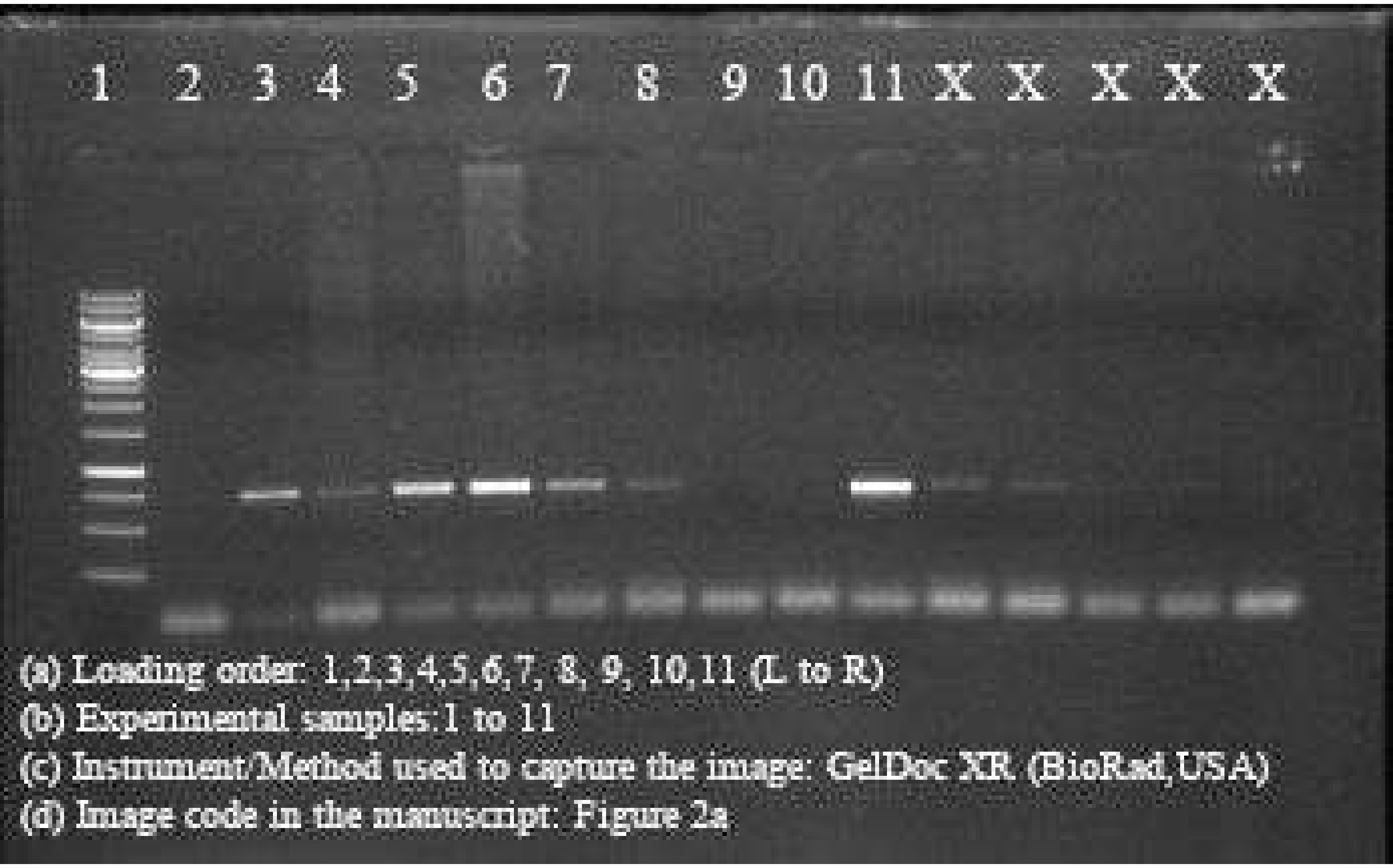
1 2 3 4 5 6 7 8 9 10 11 X X X X X
(a) Loading order: 1,2,3,4,5,6,7, 8, 9, 10,11 (L to R)
(b) Experimental samples:1 to 11
(c) Instrument/Method used to capture the image: GelDoc XR (BioRad,USA)
(d) Image code in the manuscript: Figure 2a

Raw Fig 2b

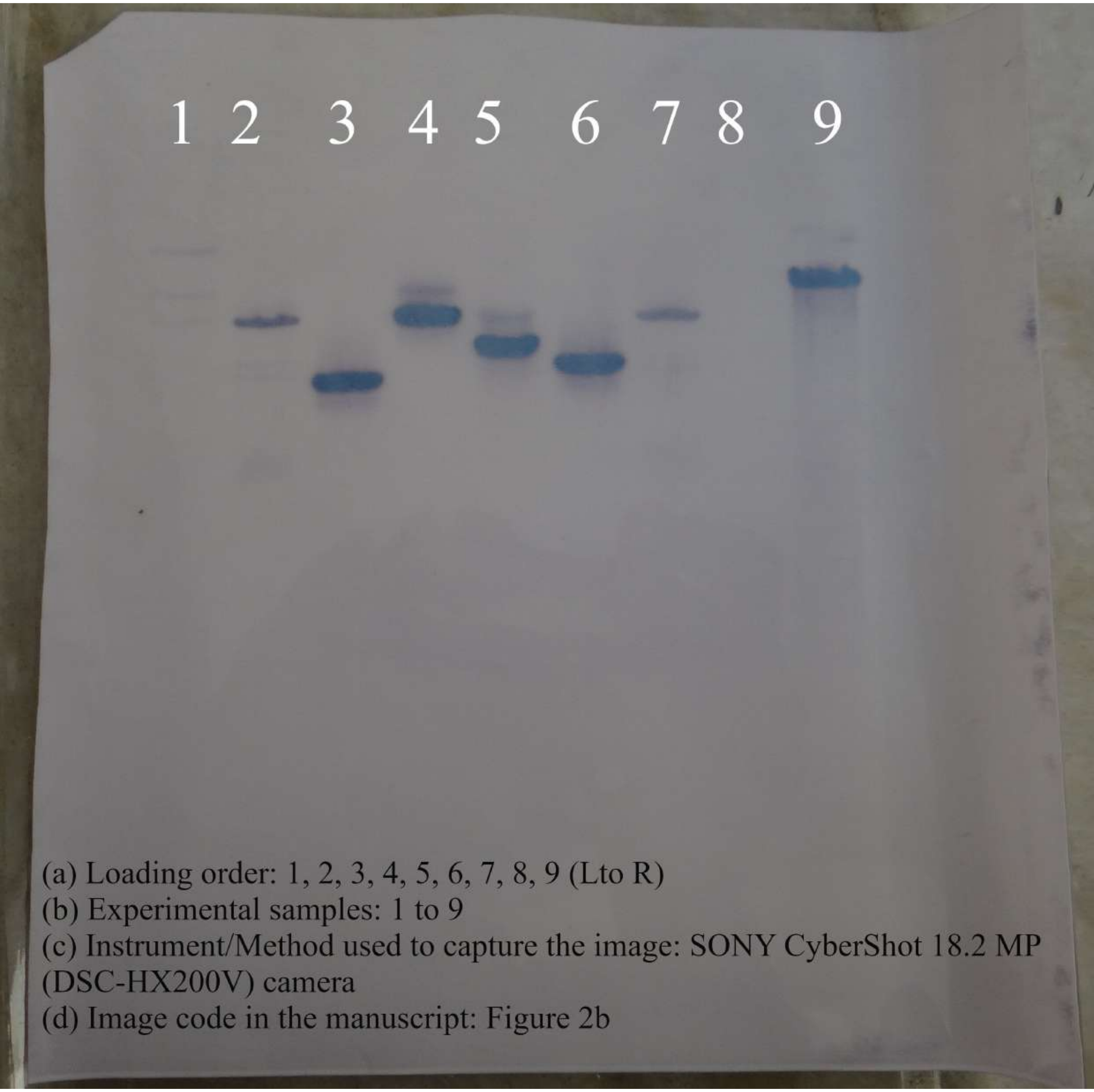

(a) Loading order: 1, 2, 3, 4, 5, 6, 7, 8, 9 (Lto R)
(b) Experimental samples: 1 to 9
(c) Instrument/Method used to capture the image: SONY CyberShot 18.2 MP (DSC-HX200V) camera
(d) Image code in the manuscript: Figure 2b

Raw Fig 3a

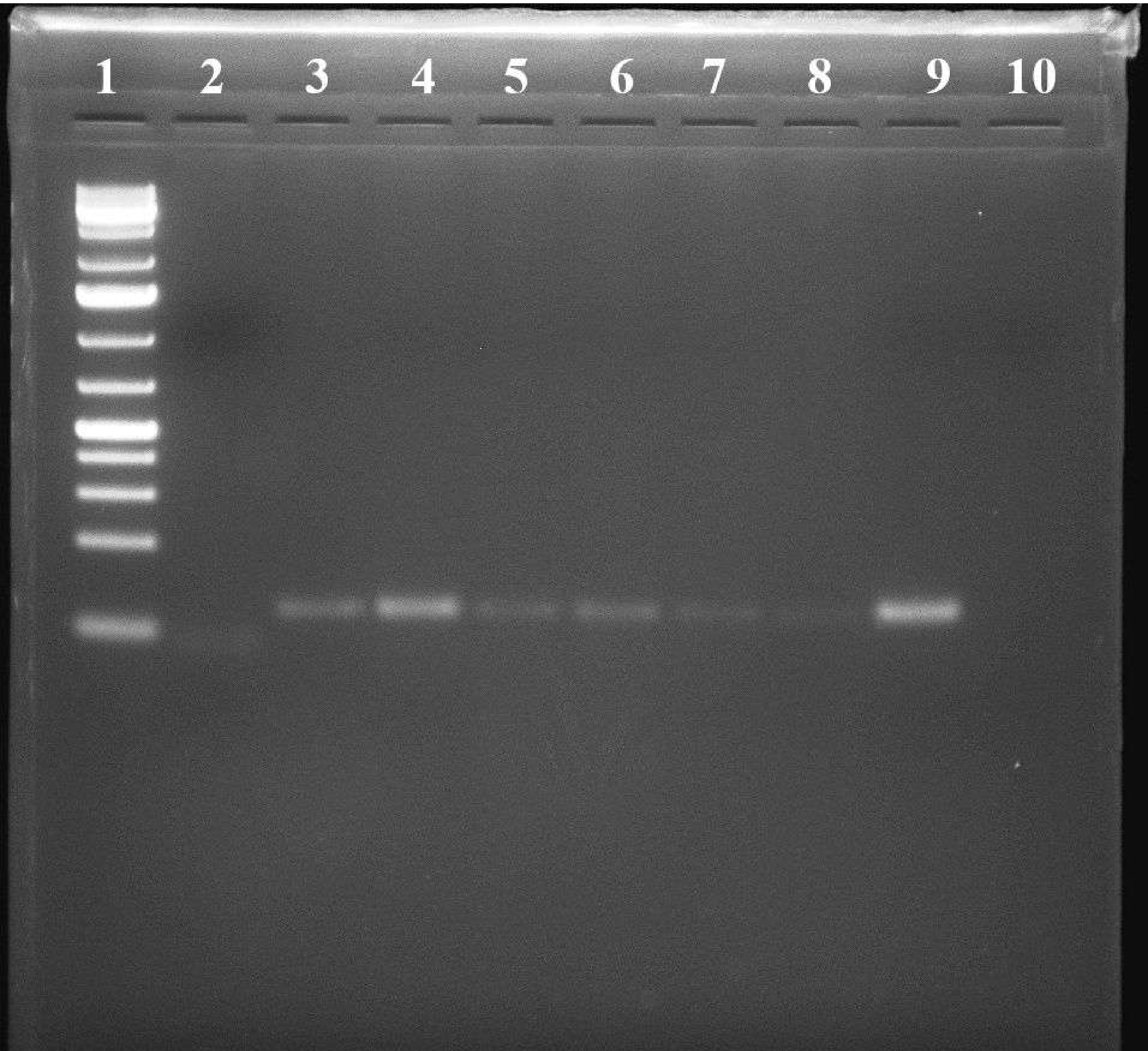

(a) Loading order: 1, 2, 3, 4, 5, 6, 7, 8, 9, 10 (L to R)
(b) Experimental samples: 1 to 10
(c) Instrument/Method used to capture the image: GelDoc XR (BioRad, USA)
(d) Image code in the manuscript: Figure 3a (Upper panel)

Raw Fig 3b

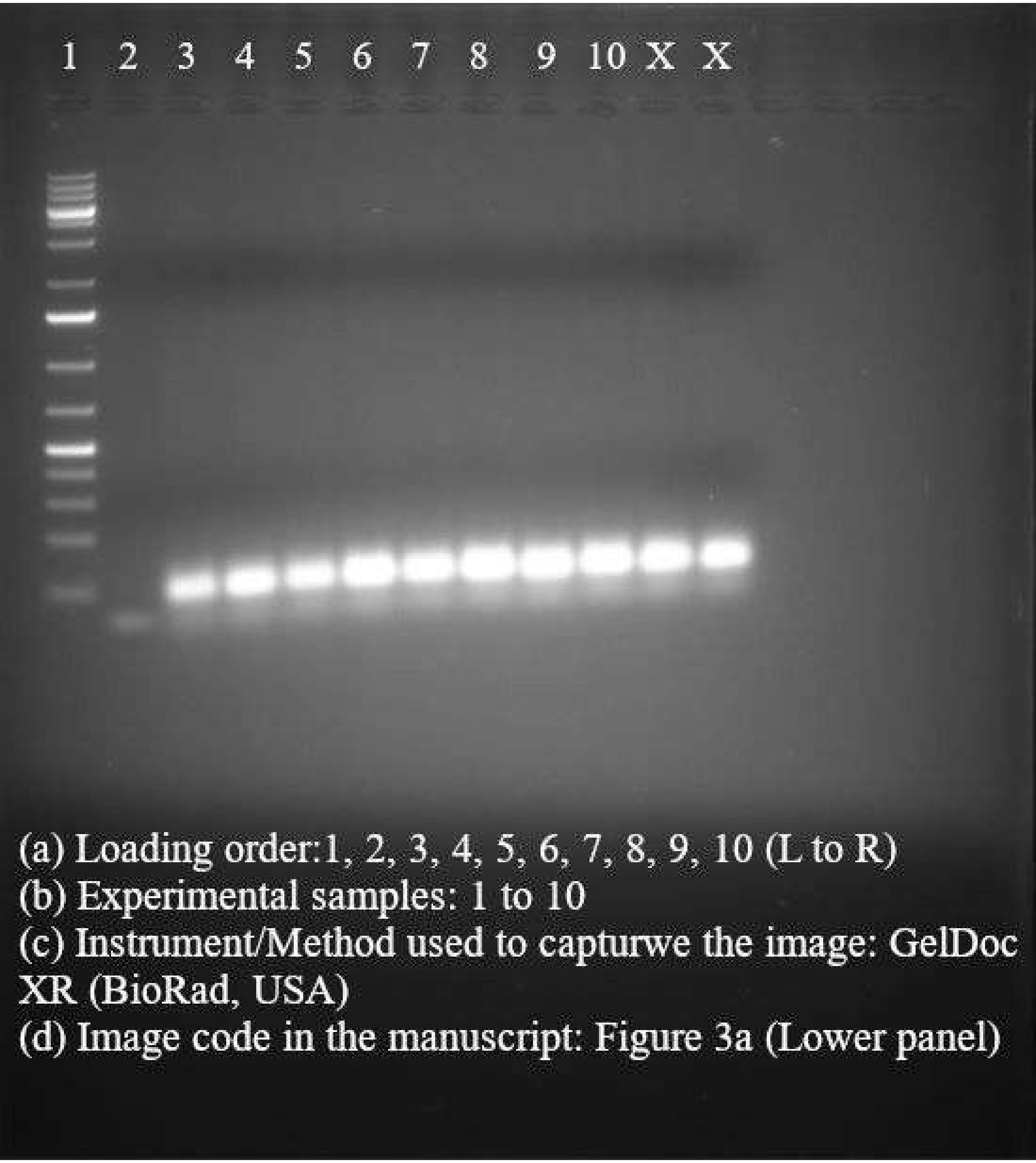
1 2 3 4 5 6 7 8 9 10 X X
(a) Loading order:1, 2, 3, 4, 5, 6, 7, 8, 9, 10 (L to R)
(b) Experimental samples: 1 to 10
(c) Instrument/Method used to capturwe the image: GelDoc XR (BioRad, USA)
(d) Image code in the manuscript: Figure 3a (Lower panel)

Raw Fig 5

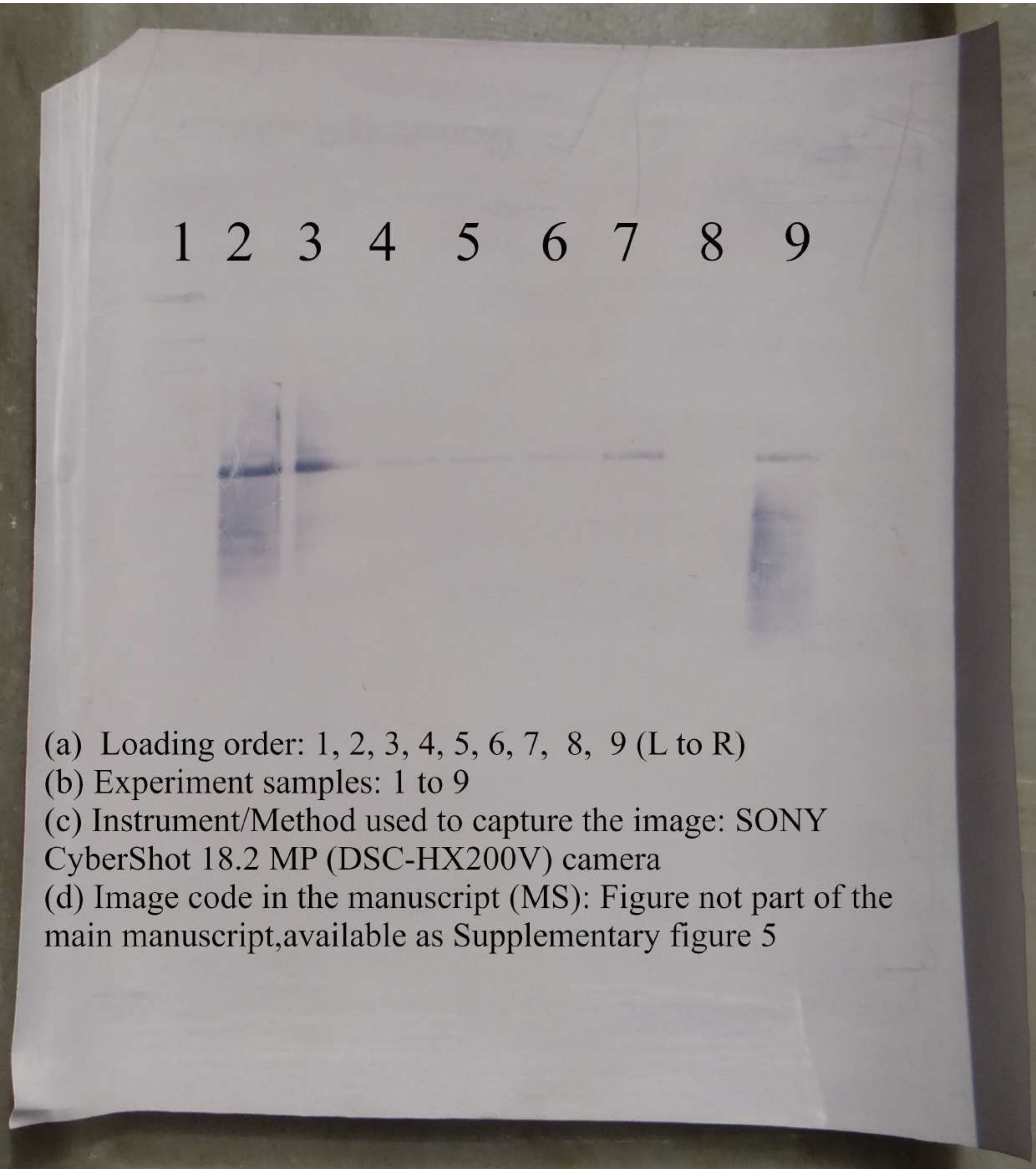
1 2 3 4 5 6 7 8 9
(a) Loading order: 1, 2, 3, 4, 5, 6, 7, 8, 9 (L to R)
(b) Experiment samples: 1 to 9
(c) Instrument/Method used to capture the image: SONY CyberShot 18.2 MP (DSC-HX200V) camera
(d) Image code in the manuscript (MS): Figure not part of the main manuscript,available as Supplementary figure 5

Raw for Supplementary Figure 4: Gel Image for PCR analysis of lines derived from VPS14.105 [L1: DNA Ladder, L2-L21: T2 progenies, L22: Control (DCP 92-3), L23: No Template Control (NTC); L24: Positive control (Recombinant Plasmid)]

Raw for Supplementary Figure 5: Gel Image for PCR analysis of lines derived from VPS47.317 [L1: DNA Ladder, L2-L18: T2 progenies, L19: Control (DCP 92-3), L20: No Template Control (NTC); L21: Positive control (Recombinant Plasmid)]

Raw for Supplementary Figure 6: Gel Image for PCR analysis of lines derived from VPS57.371 [L1: DNA Ladder, L2-L21: T2 progenies, L22: Control (DCP 92-3), L23: No Template Control (NTC); L24: Positive control (Recombinant Plasmid)]

Raw for Supplementary Figure 7: Gel Image for PCR analysis of lines derived from VPS66.405 [L1: DNA Ladder, L2-L23: T2 progenies, L24: Control (DCP 92-3), L25: No Template Control (NTC); L26: Positive control (Recombinant Plasmid)]

Raw for Supplementary Figure 8: Gel Image for PCR analysis of lines derived from VPS77.421 [L1: DNA Ladder, L2-L22: T2 progenies, L23: Control (DCP 92-3), L24: No Template Control (NTC); L25: Positive control (Recombinant Plasmid)]

Raw Image for Supplementary Figure 4

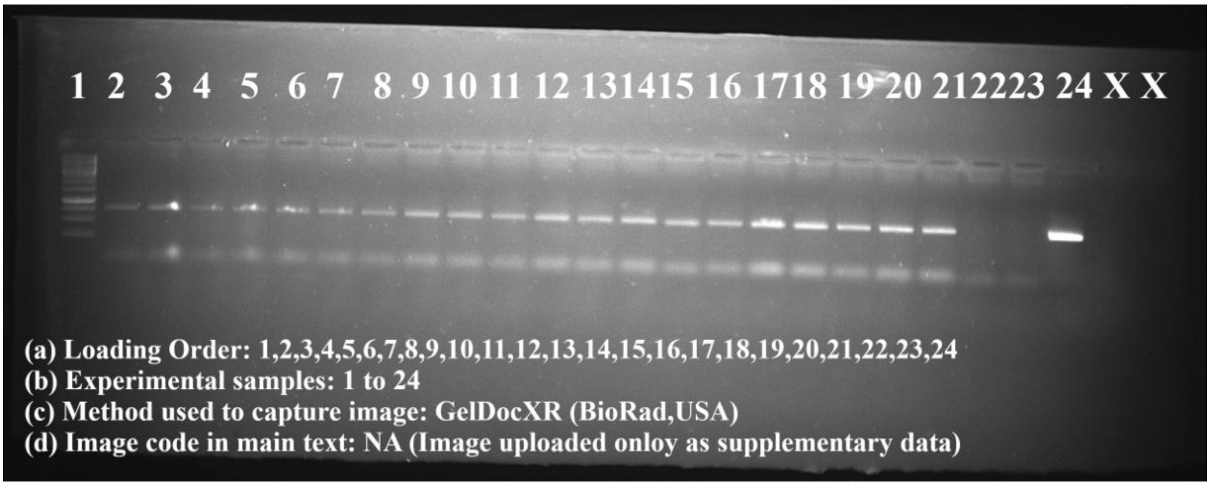

Raw Image for Supplementary Figure 5

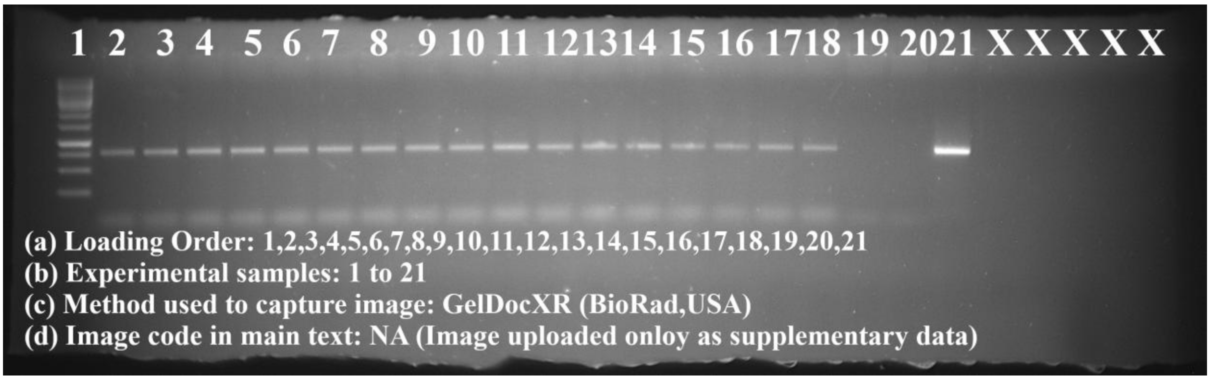

Raw Image for Supplementary Figure 6

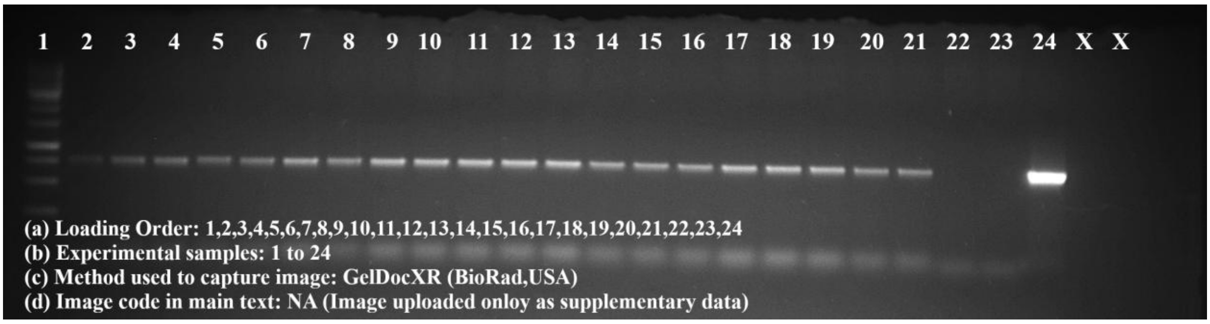

Raw Image for Supplementary Figure 7

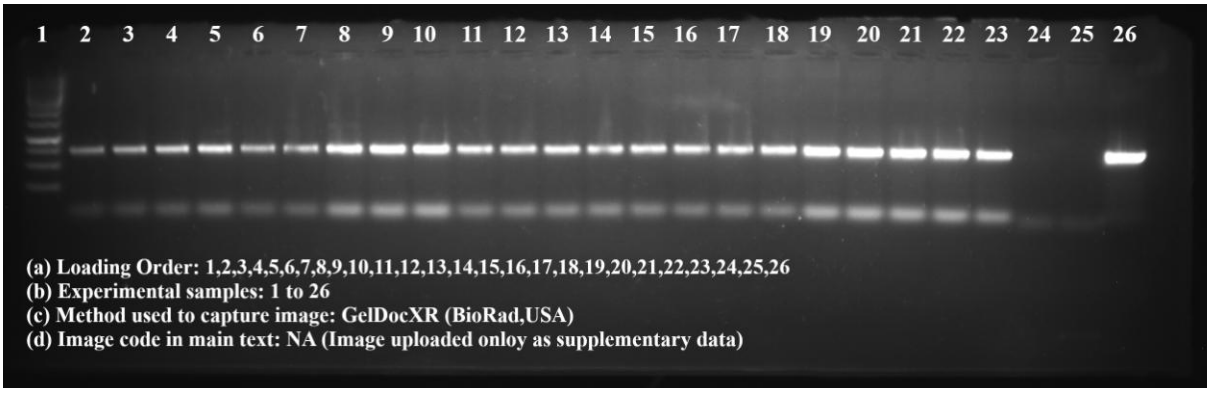

Raw Image for Supplementary Figure 8

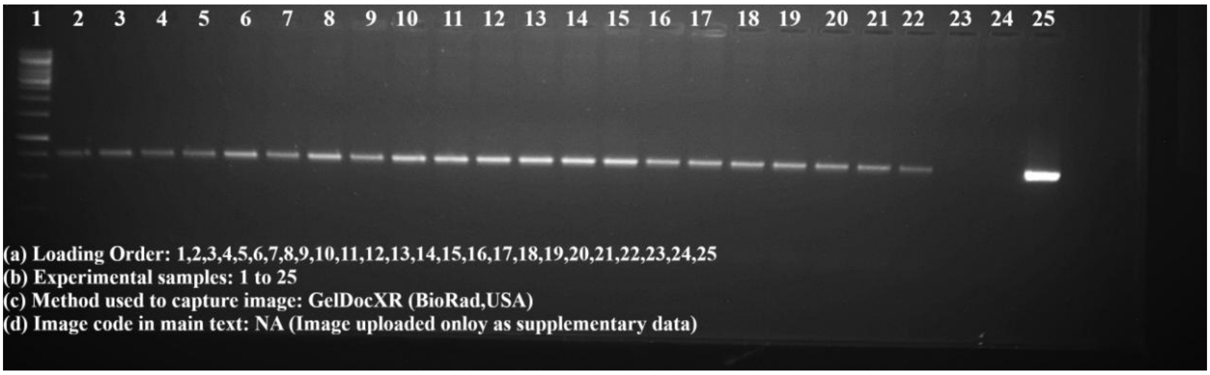

Supplement: S1 Raw images — (PDF) [file pone.0270011.s021.pdf]
