# Supplementary material for: Evaluation of transgenic chickpea harboring codon-modified Vip3Aa against gram pod borer (Helicoverpa armigera H.)
Source: PLoS One. 2022 Jun 24;17(6):e0270011. doi: 10.1371/journal.pone.0270011 (PMC9231776; doi:10.1371/journal.pone.0270011)
Supplement: S1 Info — (PDF) [file pone.0270011.s022.pdf]

> L48811.1:739-3108 *Bacillus thuringienis* strain AB88 insecticidal protein (vip3A(a) gene, complete cds

ATGAACAAGAATAATACTAAATTAAGCACAAGAGCCTTACCAAGTTTTATTGATTATTTTAATGGCATTT
ATGGATTTGCCACTGGTATCAAAGACATTATGAACATGATTTTTAAAACGGATACAGGTGGTGATCTAAC
CCTAGACGAAATTTTAAAGAATCAGCAGTTACTAAATGATATTTCTGGTAAATTGGATGGGGTGAATGGA
AGCTTAAATGATCTTATCGCACAGGGAAACTTAAATACAGAATTATCTAAGGAAATATTAAAAATTGCAA
ATGAACAAAATCAAGTTTTAAATGATGTTAATAACAAACTCGATGCGATAAATACGATGCTTCGGGTATA
TCTACCTAAAATTACCTCTATGTTGAGTGATGTAATGAAACAAAATTATGCGCTAAGTCTGCAAATAGAA
TACTTAAGTAAACAATTGCAAGAGATTTCTGATAAGTTGGATATTATTAATGTAAATGTACTTATTAACT
CTACACTTACTGAAATTACACCTGCGTATCAAAGGATTAAATATGTGAACGAAAAATTTGAGGAATTAAC
TTTTGCTACAGAAACTAGTTCAAAAGTAAAAAAGGATGGCTCTCCTGCAGATATTCTTGATGAGTTAACT
GAGTTAACTGAACTAGCGAAAAGTGTAACAAAAAATGATGTGGATGGTTTTGAATTTTACCTTAATACAT
TCCACGATGTAATGGTAGGAAATAATTTATTCGGGCGTTCAGCTTTAAAAACTGCATCGGAATTAATTAC
TAAAGAAAATGTGAAAACAAGTGGCAGTGAGGTCGGAAATGTTTATAACTTCTTAATTGTATTAACAGCT
CTGCAAGCCCAAGCTTTTCTTACTTTAACAACATGCCGAAAATTATTAGGCTTAGCAGATATTGATTATA
CTTCTATTATGAATGAACATTTAAATAAGGAAAAAGAGGAATTTAGAGTAAACATCCTCCCTACACTTTC
TAATACTTTTTCTAATCCTAATTATGCAAAAGTTAAAGGAAGTGATGAAGATGCAAAGATGATTGTGGAA
GCTAAACCAGGACATGCATTGATTGGGTTTGAAATTAGTAATGATTCAATTACAGTATTAAAAGTATATG
AGGCTAAGCTAAAACAAAATTATCAAGTCGATAAGGATTCCTTATCGGAAGTTATTTATGGTGATATGGA
TAAATTATTGTGCCCAGATCAATCTGAACAAATCTATTATACAAATAACATAGTATTTCCAAATGAATAT
GTAATTACTAAAATTGATTTCACTAAAAAAATGAAAACTTTAAGATATGAGGTAACAGCGAATTTTTATG
ATTCTTCTACAGGAGAAATTGACTTAAATAAGAAAAAAGTAGAATCAAGTGAAGCGGAGTATAGAACGTT
AAGTGCTAATGATGATGGGGTGTATATGCCGTTAGGTGTCATCAGTGAAACATTTTTGACTCCGATTAAT
GGGTTTGGCCTCCAAGCTGATGAAAATTCAAGATTAATTACTTTAACATGTAAATCATATTTAAGAGAAC
TACTGCTAGCAACAGACTTAAGCAATAAAGAAACTAAATTGATCGTCCCGCCAAGTGGTTTTATTAGCAA
TATTGTAGAGAACGGGTCCATAGAAGAGGACAATTTAGAGCCGTGGAAAGCAAATAATAAGAATGCGTAT
GTAGATCATACAGGCGGAGTGAATGGAACTAAAGCTTTATATGTTCATAAGGACGGAGGAATTTCACAAT
TTATTGGAGATAAGTTAAAACCGAAAACTGAGTATGTAATCCAATATACTGTTAAAGGAAAACCTTCTAT
TCATTTAAAAGATGAAAATACTGGATATATTCATTATGAAGATACAAATAATAATTTAGAAGATTATCAA
ACTATTAATAAACGTTTTACTACAGGAACTGATTTAAAGGGAGTGTATTTAATTTTAAAAAGTCAAAATG
GAGATGAAGCTTGGGGAGATAACTTTATTATTTTGGAAATTAGTCCTTCTGAAAAGTTATTAAGTCCAGA
ATTAATTAATACAAATAATTGGACGAGTACGGGATCAACTAATATTAGCGGTAATACACTCACTCTTTAT
CAGGGAGGACGAGGGATTCTAAAACAAAACCTTCAATTAGATAGTTTTTCAACTTATAGAGTGTATTTTT
CTGTGTCCGGAGATGCTAATGTAAGGATTAGAAATTCTAGGGAAGTGTTATTTGAAAAAAGATATATGAG
CGGTGCTAAAGATGTTTCTGAAATGTTCACTACAAAATTTGAGAAAGATAACTTTTATATAGAGCTTTCT
CAAGGGAATAATTTATATGGTGGTCCTATTGTACATTTTTACGATGTCTCTATTAAGTAA

>MZ130099.1 Synthetic construct cmVip3Aa (cmVip3Aa) gene, complete cds

ATGAACAAGAACAACACTAAGCTTTCTACTAGAGCTCTTCCATCTTTTATTGATTATTTTAATGGTATTT
ATGGTTTTGCTACTGGTATTAAGGATATTATGAACATGATTTTTAAGACTGATACTGGTGGTGATCTTAC
TCTTGATGAAATTCTTAAGAACCAACAACTTCTTAATGATATTTCTGGTAAGCTTGATGGTGTTAATGGT
TCTCTTAATGATCTTATTGCTCAAGGTAACCTTAACACTGAACTTTCTAAGGAAATTCTTAAGATTGCTA
ATGAACAAAACCAAGTTCTTAATGATGTTAACAACAAGCTTGATGCTATTAATACTATGCTTAGAGTTTA
TCTTCCAAAGATTACTTCTATGCTTTCTGATGTTATGAAGCAAAACTATGCTCTTTCTCTTCAAATTGAA
TATCTTTCTAAGCAACTTCAAGAAATTTCTGATAAGCTTGATATTATTAATGTTAATGTTCTTATTAACT
CTACTCTTACTGAAATTACTCCAGCTTATCAAAGAATTAAGTATGTTAATGAAAAGTTTGAAGAACTTAC
TTTTGCTACTGAAACTTCTTCTAAGGTTAAGAAGGATGGTTCTCCAGCTGATATTCTTGATGAACTTACT
GAACTTACTGAACTTGCTAAGTCTGTTACTAAGAATGATGTTGATGGTTTTGAATTTTATCTTAACACTT
TTCATGATGTTATGGTTGGTAACAACCTTTTTGGTAGATCTGCTCTTAAGACTGCTTCTGAACTTATTAC
TAAGGAAAATGTTAAGACTTCTGGTTCTGAAGTTGGTAATGTTTATAACTTTCTTATTGTTCTTACTGCT
CTTCAAGCTCAAGCTTTTCTTACTCTTACTACTTGTAGAAAGCTTCTTGGTCTTGCTGATATTGATTATA
CTTCTATTATGAATGAACATCTTAACAAGGAAAAGGAAGAATTTAGAGTTAACATTCTTCCAACTCTTTC
TAACACTTTTTCTAATCCAAACTATGCTAAGGTTAAGGGTTCTGATGAAGATGCTAAGATGATTGTTGAA
GCTAAGCCAGGTCATGCTCTTATTGGTTTTGAAATTTCTAATGATTCTATTACTGTTCTTAAGGTTTATG
AAGCTAAGCTTAAGCAAAACTATCAAGTTGATAAGGATTCTCTTTCTGAAGTTATTTATGGTGATATGGA
TAAGCTTCTTTGTCCAGATCAATCTGAACAAATTTATTATACTAACAACATTGTTTTTCCAAATGAATAT
GTTATTACTAAGATTGATTTTACTAAGAAGATGAAGACTCTTAGATATGAAGTTACTGCTAATTTTTATG
ATTCTTCTACTGGTGAAATTGATCTTAACAAGAAGAAGGTTGAATCTTCTGAAGCTGAGTATAGAACTCT
TTCTGCTAATGATGATGGTGTTTATATGCCACTTGGTGTTATTTCTGAAACTTTTCTTACTCCAATTAAT
GGTTTTGGTCTTCAAGCTGATGAAAATTCTAGACTTATTACTCTTACTTGTAAGTCTTATCTTAGAGAAC
TTCTTCTTGCTACTGATCTTTCTAACAAGGAAACTAAGCTTATTGTTCCACCATCTGGTTTTATTTCTAA
CATTGTTGAAAATGGTTCTATTGAAGAAGATAATCTTGAACCATGGAAGGCTAACAACAAGAACGCTTAT
GTTGATCATACTGGTGGTGTTAATGGTACTAAGGCTCTTTATGTTCATAAGGATGGTGGTATTTCTCAAT
TTATTGGTGATAAGCTTAAGCCAAAGACTGAATATGTTATTCAATATACTGTTAAGGGTAAGCCATCTAT
TCATCTTAAGGATGAAAACACTGGTTATATTCATTATGAAGATACTAACAACAACCTTGAAGATTATCAA
ACTATTAACAAGAGATTTACTACTGGTACTGATCTTAAGGGTGTTTATCTTATTCTTAAGTCTCAAAATG
GTGATGAAGCTTGGGGTGATAATTTTATTATTCTTGAAATTTCTCCATCTGAAAAGCTTCTTTCTCCAGA
ACTTATTAACACTAACAATTGGACTTCTACTGGTTCTACTAACATTTCTGGTAACACTCTTACTCTTTAT
CAAGGTGGTAGAGGTATTCTTAAGCAAAATCTTCAACTTGATTCTTTTTCTACTTATAGAGTTTATTTTT
CTGTTTCTGGTGATGCTAATGTTAGAATTAGAAATTCTAGAGAAGTTCTTTTTGAAAAGAGATATATGTC
TGGTGCTAAGGATGTTTCTGAAATGTTTACTACTAAGTTTGAAAAGGATAATTTTTATATTGAACTTTCT
CAAGGTAACAACCTTTATGGTGGTCCAATTGTTCATTTTTATGATGTTTCTATTAAGTAA

```
CLUSTAL O(1.2.4) multiple sequence alignment


L48811.1        ATGAACAAGAATAATACTAAATTAAGCACAAGAGCCTTACCAAGTTTTATTGATTATTTT    60
MZ130099.1      ATGAACAAGAACAACACTAAGCTTTCTACTAGAGCTCTTCCATCTTTTATTGATTATTTT    60
                *********** ** *****  *    ** *****  * ***  ****************

L48811.1        AATGGCATTTATGGATTTGCCACTGGTATCAAAGACATTATGAACATGATTTTTAAAACG    120
MZ130099.1      AATGGTATTTATGGTTTTGCTACTGGTATTAAGGATATTATGAACATGATTTTTAAGACT    120
                ***** ******** ***** ******** ** ** ******************** **

L48811.1        GATACAGGTGGTGATCTAACCCTAGACGAAATTTTAAAGAATCAGCAGTTACTAAATGAT    180
MZ130099.1      GATACTGGTGGTGATCTTACTCTTGATGAAATTCTTAAGAACCAACAACTTCTTAATGAT    180
                ***** *********** ** ** ** ****** * ***** ** **  * ** ******

L48811.1        ATTTCTGGTAAATTGGATGGGGTGAATGGAAGCTTAAATGATCTTATCGCACAGGGAAAC    240
MZ130099.1      ATTTCTGGTAAGCTTGATGGTGTTAATGGTTCTCTTAATGATCTTATTGCTCAAGGTAAC    240
                ***********  * ***** ** *****     * *********** ** ** ** ***

L48811.1        TTAAATACAGAATTATCTAAGGAAATATTAAAAATTGCAAATGAACAAAATCAAGTTTTA    300
MZ130099.1      CTTAACACTGAACTTTCTAAGGAAATTCTTAAGATTGCTAATGAACAAAACCAAGTTCTT    300
                 * ** ** *** * ***********  * ** ***** *********** ****** *

L48811.1        AATGATGTTAATAACAAACTCGATGCGATAAATACGATGCTTCGGGTATATCTACCTAAA    360
MZ130099.1      AATGATGTTAACAACAAGCTTGATGCTATTAATACTATGCTTAGAGTTTATCTTCCAAAG    360
                *********** ***** ** ***** ** ***** ****** * ** ***** ** **

L48811.1        ATTACCTCTATGTTGAGTGATGTAATGAAACAAAATTATGCGCTAAGTCTGCAAATAGAA    420
MZ130099.1      ATTACTTCTATGCTTTCTGATGTTATGAAGCAAAACTATGCTCTTTCTCTTCAAATTGAA    420
                ***** ****** *   ****** ***** ***** ***** **   *** ***** ***

L48811.1        TACTTAAGTAAACAATTGCAAGAGATTTCTGATAAGTTGGATATTATTAATGTAAATGTA    480
MZ130099.1      TATCTTTCTAAGCAACTTCAAGAAATTTCTGATAAGCTTGATATTATTAATGTTAATGTT    480
                **  *   *** *** * ***** ************ * ************** *****

L48811.1        CTTATTAACTCTACACTTACTGAAATTACACCTGCGTATCAAAGGATTAAATATGTGAAC    540
MZ130099.1      CTTATTAACTCTACTCTTACTGAAATTACTCCAGCTTATCAAAGAATTAAGTATGTTAAT    540
                ************** ************** ** ** ******** ***** ***** **

L48811.1        GAAAAATTTGAGGAATTAACTTTTGCTACAGAAACTAGTTCAAAAGTAAAAAAGGATGGC    600
MZ130099.1      GAAAAGTTTGAAGAACTTACTTTTGCTACTGAAACTTCTTCTAAGGTTAAGAAGGATGGT    600
                ***** ***** *** * *********** ******  *** ** ** ** ********

L48811.1        TCTCCTGCAGATATTCTTGATGAGTTAACTGAGTTAACTGAACTAGCGAAAAGTGTAACA    660
MZ130099.1      TCTCCAGCTGATATTCTTGATGAACTTACTGAACTTACTGAACTTGCTAAGTCTGTTACT    660
                ***** ** **************  * *****  * ******** ** **   *** **

L48811.1        AAAAATGATGTGGATGGTTTTGAATTTTACCTTAATACATTCCACGATGTAATGGTAGGA    720
MZ130099.1      AAGAATGATGTTGATGGTTTTGAATTTTATCTTAACACTTTTCATGATGTTATGGTTGGT    720
                ** ******** ***************** ***** ** ** ** ***** ***** **

L48811.1        AATAATTTATTCGGGCGTTCAGCTTTAAAAACTGCATCGGAATTAATTACTAAAGAAAAT    780
MZ130099.1      AACAACCTTTTTGGTAGATCTGCTCTTAAGACTGCTTCTGAACTTATTACTAAGGAAAAT    780
                ** **  * ** **  * ** *** * ** ***** ** *** * ******** ******

L48811.1        GTGAAAACAAGTGGCAGTGAGGTCGGAAATGTTTATAACTTCTTAATTGTATTAACAGCT    840
MZ130099.1      GTTAAGACTTCTGGTTCTGAAGTTGGTAATGTTTATAACTTTCTTATTGTTCTTACTGCT    840
                ** ** **   ***   *** ** ** **************  * *****  * ** ***

L48811.1        CTGCAAGCCCAAGCTTTTCTTACTTTAACAACATGCCGAAAATTATTAGGCTTAGCAGAT    900
MZ130099.1      CTTCAAGCTCAAGCTTTTCTTACTCTTACTACTTGTAGAAAGCTTCTTGGTCTTGCTGAT    900
                ** ***** *************** * ** ** **  ****  *  * **  * ** ***

L48811.1        ATTGATTATACTTCTATTATGAATGAACATTTAAATAAGGAAAAAGAGGAATTTAGAGTA    960
MZ130099.1      ATTGATTATACTTCTATTATGAATGAACATCTTAACAAGGAAAAGGAAGAATTTAGAGTT    960
                ****************************** * ** ******** ** *********

L48811.1        AACATCCTCCCTACACTTTCTAATACTTTTTCTAATCCTAATTATGCAAAAGTTAAAGGA    1020
```

```
MZ130099.1      AACATTCTTCCAACTCTTTCTAACACTTTTTCTAATCCAAACTATGCTAAGGTTAAGGGT    1020
                ***** ** ** ** ******** ************** ** ***** ** ***** ** 

L48811.1        AGTGATGAAGATGCAAAGATGATTGTGGAAGCTAAACCAGGACATGCATTGATTGGGTTT    1080
MZ130099.1      TCTGATGAAGATGCTAAGATGATTGTTGAAGCTAAGCCAGGTCATGCTCTTATTGGTTTT    1080
                  ************ *********** ******** ***** *****  * ***** ***

L48811.1        GAAATTAGTAATGATTCAATTACAGTATTAAAAGTATATGAGGCTAAGCTAAAACAAAAT    1140
MZ130099.1      GAAATTTCTAATGATTCTATTACTGTTCTTAAGGTTTATGAAGCTAAGCTTAAGCAAAAC    1140
                ******  ********* ***** **  * ** ** ***** ******** ** ***** 

L48811.1        TATCAAGTCGATAAGGATTCCTTATCGGAAGTTATTTATGGTGATATGGATAAATTATTG    1200
MZ130099.1      TATCAAGTTGATAAGGATTCTCTTTCTGAAGTTATTTATGGTGATATGGATAAGCTTCTT    1200
                ******** ***********  * ** **************************  *  * 

L48811.1        TGCCCAGATCAATCTGAACAAATCTATTATACAAATAACATAGTATTTCCAAATGAATAT    1260
MZ130099.1      TGTCCAGATCAATCTGAACAAATTTATTATACTAACAACATTGTTTTTCCAAATGAATAT    1260
                ** ******************** ******** ** ***** ** ***************

L48811.1        GTAATTACTAAAATTGATTTCACTAAAAAAATGAAAACTTTAAGATATGAGGTAACAGCG    1320
MZ130099.1      GTTATTACTAAGATTGATTTTACTAAGAAGATGAAGACTCTTAGATATGAAGTTACTGCT    1320
                ** ******** ******** ***** ** ***** *** * ******** ** ** ** 

L48811.1        AATTTTTATGATTCTTCTACAGGAGAAATTGACTTAAATAAGAAAAAAGTAGAATCAAGT    1380
MZ130099.1      AATTTTTATGATTCTTCTACTGGTGAAATTGATCTTAACAAGAAGAAGGTTGAATCTTCT    1380
                ******************** ** ********  * ** ***** ** ** *****   *

L48811.1        GAAGCGGAGTATAGAACGTTAAGTGCTAATGATGATGGGGTGTATATGCCGTTAGGTGTC    1440
MZ130099.1      GAAGCTGAGTATAGAACTCTTTCTGCTAATGATGATGGTGTTTATATGCCACTTGGTGTT    1440
                ***** ***********  *   *************** ** ********  * ***** 

L48811.1        ATCAGTGAAACATTTTTGACTCCGATTAATGGGTTTGGCCTCCAAGCTGATGAAAATTCA    1500
MZ130099.1      ATTTCTGAAACTTTTCTTACTCCAATTAATGGTTTTGGTCTTCAAGCTGATGAAAATTCT    1500
                **   ****** *** * ***** ******** ***** ** ***************** 

L48811.1        AGATTAATTACTTTAACATGTAAATCATATTTAAGAGAACTACTGCTAGCAACAGACTTA    1560
MZ130099.1      AGACTTATTACTCTTACTTGTAAGTCTTATCTTAGAGAACTTCTTCTTGCTACTGATCTT    1560
                *** * ****** * ** ***** ** *** * ******** ** ** ** ** **  * 

L48811.1        AGCAATAAAGAAACTAAATTGATCGTCCCGCCAAGTGGTTTTATTAGCAATATTGTAGAG    1620
MZ130099.1      TCTAACAAGGAAACTAAGCTTATTGTTCCACCATCTGGTTTTATTTCTAACATTGTTGAA    1620
                   ** ** ********  * ** ** ** ***  **********   ** ***** ** 

L48811.1        AACGGGTCCATAGAAGAGGACAATTTAGAGCCGTGGAAAGCAAATAATAAGAATGCGTAT    1680
MZ130099.1      AATGGTTCTATTGAAGAAGATAATCTTGAACCATGGAAGGCTAACAACAAGAACGCTTAT    1680
                ** ** ** ** ***** ** *** * ** ** ***** ** ** ** ***** ** ***

L48811.1        GTAGATCATACAGGCGGAGTGAATGGAACTAAAGCTTTATATGTTCATAAGGACGGAGGA    1740
MZ130099.1      GTTGATCATACTGGTGGTGTTAATGGTACTAAGGCTCTTTATGTTCATAAGGATGGTGGT    1740
                ** ******** ** ** ** ***** ***** *** * ************** ** ** 

L48811.1        ATTTCACAATTTATTGGAGATAAGTTAAAACCGAAAACTGAGTATGTAATCCAATATACT    1800
MZ130099.1      ATTTCTCAATTTATTGGTGATAAGCTTAAGCCAAAGACTGAATATGTTATTCAATATACT    1800
                ***** *********** ****** * ** ** ** ***** ***** ** *********

L48811.1        GTTAAAGGAAAACCTTCTATTCATTTAAAAGATGAAAATACTGGATATATTCATTATGAA    1860
MZ130099.1      GTTAAGGGTAAGCCATCTATTCATCTTAAGGATGAAAACACTGGTTATATTCATTATGAA    1860
                ***** ** ** ** ********* * ** ******** ***** ***************

L48811.1        GATACAAATAATAATTTAGAAGATTATCAAACTATTAATAAACGTTTTACTACAGGAACT    1920
MZ130099.1      GATACTAACAACAACCTTGAAGATTATCAAACTATTAACAAGAGATTTACTACTGGTACT    1920
                ***** ** ** **  * ******************** **  * ******** ** ***

L48811.1        GATTTAAAGGGAGTGTATTTAATTTTAAAAAGTCAAAATGGAGATGAAGCTTGGGGAGAT    1980
MZ130099.1      GATCTTAAGGGTGTTTATCTTATTCTTAAGTCTCAAAATGGTGATGAAGCTTGGGGTGAT    1980
                *** * ***** ** *** * *** * **   ********* ************** ***

L48811.1        AACTTTATTATTTTGGAAATTAGTCCTTCTGAAAAGTTATTAAGTCCAGAATTAATTAAT    2040
MZ130099.1      AATTTTATTATTCTTGAAATTTCTCCATCTGAAAAGCTTCTTTCTCCAGAACTTATTAAC    2040
```

```
                ** ********* * ******  *** ********* *  *   ******* * *****

L48811.1        ACAAATAATTGGACGAGTACGGGATCAACTAATATTAGCGGTAATACACTCACTCTTTAT    2100
MZ130099.1      ACTAACAATTGGACTTCTACTGGTTCTACTAACATTTCTGGTAACACTCTTACTCTTTAT    2100
                ** ** ********   *** ** ** ***** ***   ***** ** ** *********

L48811.1        CAGGGAGGACGAGGGATTCTAAAACAAAACCTTCAATTAGATAGTTTTTCAACTTATAGA    2160
MZ130099.1      CAAGGTGGTAGAGGTATTCTTAAGCAAAATCTTCAACTTGATTCTTTTTCTACTTATAGA    2160
                ** ** **  **** ***** ** ***** ****** * ***  ****** ********

L48811.1        GTGTATTTTTCTGTGTCCGGAGATGCTAATGTAAGGATTAGAAATTCTAGGGAAGTGTTA    2220
MZ130099.1      GTTTATTTTTCTGTTTCTGGTGATGCTAATGTTAGAATTAGAAATTCTAGAGAAGTTCTT    2220
                ** *********** ** ** *********** ** ************** *****  *

L48811.1        TTTGAAAAAAGATATATGAGCGGTGCTAAAGATGTTTCTGAAATGTTCACTACAAAATTT    2280
MZ130099.1      TTTGAAAAGAGATATATGTCTGGTGCTAAGGATGTTTCTGAAATGTTTACTACTAAGTTT    2280
                ******** *********   ******** **************** ***** ** ***

L48811.1        GAGAAAGATAACTTTTATATAGAGCTTTCTCAAGGGAATAATTTATATGGTGGTCCTATT    2340
MZ130099.1      GAAAAGGATAATTTTTATATTGAACTTTCTCAAGGTAACAACCTTTATGGTGGTCCAATT    2340
                ** ** ***** ******** ** *********** ** **  * *********** ***

L48811.1        GTACATTTTTACGATGTCTCTATTAAGTAA  2370
MZ130099.1      GTTCATTTTTATGATGTTTCTATTAAGTAA  2370
                ** ******** ***** ************
```
