# Supplementary material for: Evaluation of transgenic chickpea harboring codon-modified Vip3Aa against gram pod borer (Helicoverpa armigera H.)
Source: PLoS One. 2022 Jun 24;17(6):e0270011. doi: 10.1371/journal.pone.0270011 (PMC9231776; doi:10.1371/journal.pone.0270011)
Supplement: S2 Info — (PDF) [file pone.0270011.s023.pdf]

>AAC37036.1 Vip3A(a) [Bacillus thuringiensis]

MNKNNTKLSTRALPSFIDYFNGIYGFATGIKDIMNMIFKTDTGGDLTLDEILKNQQLLNDISGKLDGVNG
SLNDLIAQGNLNTELSKEILKIANEQNQVLNDVNNKLDAINTMLRVYLPKITSMLSDVMKQNYALSLQIE
YLSKQLQEISDKLDIINVNVLINSTLTEITPAYQRIKYVNEKFEELTFATETSSKVKKDGSPADILDELT
ELTELAKSVTKNDVDGFEFYLNTFHDVMVGNNLFGRSALKTASELITKENVKTSGSEVGNVYNFLIVLTA
LQAQAFLTLTTCRKLLGLADIDYTSIMNEHLNKEKEEFRVNILPTLSNTFSNPNYAKVKGSDEDAKMIVE
AKPGHALIGFEISNDSITVLKVYEAKLKQNYQVDKDSLSEVIYGDMDKLLCPDQSEQIYYTNNIVFPNEY
VITKIDFTKKMKTLRYEVTANFYDSSTGEIDLNKKKVESSEAEYRTLSANDDGVYMPLGVISETFLTPIN
GFGLQADENSRLITLTCKSYLRELLLATDLSNKETKLIVPPSGFISNIVENGSIEEDNLEPWKANNKNAY
VDHTGGVNGTKALYVHKDGGISQFIGDKLKPKTEYVIQYTVKGKPSIHLKDENTGYIHYEDTNNNLEDYQ
TINKRFTTGTDLKGVYLILKSQNGDEAWGDNFIILEISPSEKLLSPELINTNNWTSTGSTNISGNTLTLY
QGGRGILKQNLQLDSFSTYRVYFSVSGDANVRIRNSREVLFEKRYMSGAKDVSEMFTTKFEKDNFYIELS
QGNNLYGGPIVHFYDVSIK

>QVQ68825.1 cmVip3Aa [synthetic construct]

MNKNNTKLSTRALPSFIDYFNGIYGFATGIKDIMNMIFKTDTGGDLTLDEILKNQQLLNDISGKLDGVNG
SLNDLIAQGNLNTELSKEILKIANEQNQVLNDVNNKLDAINTMLRVYLPKITSMLSDVMKQNYALSLQIE
YLSKQLQEISDKLDIINVNVLINSTLTEITPAYQRIKYVNEKFEELTFATETSSKVKKDGSPADILDELT
ELTELAKSVTKNDVDGFEFYLNTFHDVMVGNNLFGRSALKTASELITKENVKTSGSEVGNVYNFLIVLTA
LQAQAFLTLTTCRKLLGLADIDYTSIMNEHLNKEKEEFRVNILPTLSNTFSNPNYAKVKGSDEDAKMIVE
AKPGHALIGFEISNDSITVLKVYEAKLKQNYQVDKDSLSEVIYGDMDKLLCPDQSEQIYYTNNIVFPNEY
VITKIDFTKKMKTLRYEVTANFYDSSTGEIDLNKKKVESSEAEYRTLSANDDGVYMPLGVISETFLTPIN
GFGLQADENSRLITLTCKSYLRELLLATDLSNKETKLIVPPSGFISNIVENGSIEEDNLEPWKANNKNAY
VDHTGGVNGTKALYVHKDGGISQFIGDKLKPKTEYVIQYTVKGKPSIHLKDENTGYIHYEDTNNNLEDYQ
TINKRFTTGTDLKGVYLILKSQNGDEAWGDNFIILEISPSEKLLSPELINTNNWTSTGSTNISGNTLTLY
QGGRGILKQNLQLDSFSTYRVYFSVSGDANVRIRNSREVLFEKRYMSGAKDVSEMFTTKFEKDNFYIELS
QGNNLYGGPIVHFYDVSIK

```
CLUSTAL O(1.2.4) multiple sequence alignment


AAC37036.1      MNKNNTKLSTRALPSFIDYFNGIYGFATGIKDIMNMIFKTDTGGDLTLDEILKNQQLLND    60
QVQ68825.1      MNKNNTKLSTRALPSFIDYFNGIYGFATGIKDIMNMIFKTDTGGDLTLDEILKNQQLLND    60
                ************************************************************

AAC37036.1      ISGKLDGVNGSLNDLIAQGNLNTELSKEILKIANEQNQVLNDVNNKLDAINTMLRVYLPK    120
QVQ68825.1      ISGKLDGVNGSLNDLIAQGNLNTELSKEILKIANEQNQVLNDVNNKLDAINTMLRVYLPK    120
                ************************************************************

AAC37036.1      ITSMLSDVMKQNYALSLQIEYLSKQLQEISDKLDIINVNVLINSTLTEITPAYQRIKYVN    180
QVQ68825.1      ITSMLSDVMKQNYALSLQIEYLSKQLQEISDKLDIINVNVLINSTLTEITPAYQRIKYVN    180
                ************************************************************

AAC37036.1      EKFEELTFATETSSKVKKDGSPADILDELTELTELAKSVTKNDVDGFEFYLNTFHDVMVG    240
QVQ68825.1      EKFEELTFATETSSKVKKDGSPADILDELTELTELAKSVTKNDVDGFEFYLNTFHDVMVG    240
                ************************************************************

AAC37036.1      NNLFGRSALKTASELITKENVKTSGSEVGNVYNFLIVLTALQAQAFLTLTTCRKLLGLAD    300
QVQ68825.1      NNLFGRSALKTASELITKENVKTSGSEVGNVYNFLIVLTALQAQAFLTLTTCRKLLGLAD    300
                ************************************************************

AAC37036.1      IDYTSIMNEHLNKEKEEFRVNILPTLSNTFSNPNYAKVKGSDEDAKMIVEAKPGHALIGF    360
QVQ68825.1      IDYTSIMNEHLNKEKEEFRVNILPTLSNTFSNPNYAKVKGSDEDAKMIVEAKPGHALIGF    360
                ************************************************************

AAC37036.1      EISNDSITVLKVYEAKLKQNYQVDKDSLSEVIYGDMDKLLCPDQSEQIYYTNNIVFPNEY    420
QVQ68825.1      EISNDSITVLKVYEAKLKQNYQVDKDSLSEVIYGDMDKLLCPDQSEQIYYTNNIVFPNEY    420
                ************************************************************

AAC37036.1      VITKIDFTKKMKTLRYEVTANFYDSSTGEIDLNKKKVESSEAEYRTLSANDDGVYMPLGV    480
QVQ68825.1      VITKIDFTKKMKTLRYEVTANFYDSSTGEIDLNKKKVESSEAEYRTLSANDDGVYMPLGV    480
                ************************************************************

AAC37036.1      ISETFLTPINGFGLQADENSRLITLTCKSYLRELLLATDLSNKETKLIVPPSGFISNIVE    540
QVQ68825.1      ISETFLTPINGFGLQADENSRLITLTCKSYLRELLLATDLSNKETKLIVPPSGFISNIVE    540
                ************************************************************

AAC37036.1      NGSIEEDNLEPWKANNKNAYVDHTGGVNGTKALYVHKDGGISQFIGDKLKPKTEYVIQYT    600
QVQ68825.1      NGSIEEDNLEPWKANNKNAYVDHTGGVNGTKALYVHKDGGISQFIGDKLKPKTEYVIQYT    600
                ************************************************************

AAC37036.1      VKGKPSIHLKDENTGYIHYEDTNNNLEDYQTINKRFTTGTDLKGVYLILKSQNGDEAWGD    660
QVQ68825.1      VKGKPSIHLKDENTGYIHYEDTNNNLEDYQTINKRFTTGTDLKGVYLILKSQNGDEAWGD    660
                ************************************************************

AAC37036.1      NFIILEISPSEKLLSPELINTNNWTSTGSTNISGNTLTLYQGGRGILKQNLQLDSFSTYR    720
QVQ68825.1      NFIILEISPSEKLLSPELINTNNWTSTGSTNISGNTLTLYQGGRGILKQNLQLDSFSTYR    720
                ************************************************************

AAC37036.1      VYFSVSGDANVRIRNSREVLFEKRYMSGAKDVSEMFTTKFEKDNFYIELSQGNNLYGGPI    780
QVQ68825.1      VYFSVSGDANVRIRNSREVLFEKRYMSGAKDVSEMFTTKFEKDNFYIELSQGNNLYGGPI    780
                ************************************************************

AAC37036.1      VHFYDVSIK       789
QVQ68825.1      VHFYDVSIK       789
                *********
```
